# Life Course Exposure to Cyanobacteria and Amyotrophic Lateral Sclerosis Survival

**DOI:** 10.3390/ijerph22050763

**Published:** 2025-05-12

**Authors:** Stuart A. Batterman, Md Kamrul Islam, Dae Gyu Jang, Eva L. Feldman, Stephen A. Goutman

**Affiliations:** 1 Department of Environmental Health Sciences, University of Michigan, Ann Arbor, MI 48109, USA; kamrul@umich.edu; 2 Department of Neurology, University of Michigan, Ann Arbor, MI 48109, USA; dajang@med.umich.edu (D.G.J.); efeldman@umich.edu (E.L.F.);; 3 NeuroNetwork for Emerging Therapies, University of Michigan, Ann Arbor, MI 48109, USA

**Keywords:** neurological degenerative disease, harmful algae bloom, cyanobacteria, survival, geostatistics, residence, water, environmental exposure

## Abstract

Cyanobacterial harmful algal blooms (cyanoHABs) occur worldwide and can cause ingestion and inhalation exposure to microcystin and other potent toxins. This study develops life course exposure measures for cyanobacteria for application in population studies and then associates these measures with the survival of individuals with amyotrophic lateral sclerosis (ALS). The exposure measures utilize an individual’s residence history, date of disease onset, and satellite data from the Cyanobacteria Assessment Network. Residence duration for selected exposure windows referenced to disease onset date was used to weight cyanobacteria concentrations in water bodies within 0.25 to 10 km of each residence. Different concentration metrics, buffer sizes, and exposure windows were evaluated. The 2.5 and 5 km buffers best balanced the likelihood and plausibility of exposure while still resolving exposure contrasts. Over their lifetime, most study participants lived within 5 km of cyanobacteria blooms, and the exposure was associated with up to 0.89 years shorter survival, with significant interactions for individuals reporting swimming, fishing, and private wells. Our findings suggest a new and modifiable risk factor for ALS survival, and a need to confirm exposures and epidemiological findings. These cyanoHAB exposure estimates can facilitate population studies that can discover new relationships with neurodegenerative and other diseases.

## 1. Introduction

Cyanobacterial harmful algal blooms (cyanoHABs) occur in freshwater, marine, and terrestrial ecosystems worldwide and are of concern due to potential health, ecological, taste, and odor impacts and increasing bloom intensity, due in part to climate change [1,2,3,4]. Cyanobacteria produce a wide range of genotoxic and neurotoxic agents including hepatotoxins, neurotoxins, dermatotoxins, and cytotoxins, e.g., microcystins and ß-methylamino-L-alanine (BMAA) [5,6,7]. The frequency and nature of cyanoHAB events depend on many factors, including the watershed, weather, and nutrient loading. Blooms differ greatly in size (from localized shoreline accumulations to large areas in the oceans and the Great Lakes), depth (planktonic to benthic), duration (hours to weeks), composition, and concentration of cyanobacteria toxins. While the World Health Organization (WHO) has established concentration guidelines intended to be protective of acute adverse human, animal and ecological effects (20,000 and 100,000 cells/mL for moderate and high risk, respectively) [8], the understanding of exposures and impacts of cyanoHAB on human health remains limited [9,10,11].

A few population-level studies have examined linkages between cyanoHABs and the risk of disease. CyanoHAB-related exposures have been associated with liver disease in the US [12,13], Korea [14] and China [15]; brain and other cancers in Serbia [16]; and amyotrophic lateral sclerosis (ALS) in 14 different studies in the US, France, Guadeloupe, Italy and Japan [6]. In most studies, cyanoHAB exposure was based on measurements in public drinking water supplies or food, although indirect measures using remote sensing (satellite) data have been used in several instances, e.g., at county or 4 to 8 km scales using a single time point [13,17]. Exposure to cyanobacteria toxins can occur via ingestion, inhalation, and dermal contact from drinking water, recreational water activities, aerosols, food, medicinal water (e.g., hemodialysis), showering and bathing, washing utensils and other objects, and skin irritation [5,18]. While largely unexamined, the inhalation route may be important given evidence that cyanoHAB toxins can be airborne [9,19] and that nasal exposure may increase toxicity from rapid uptake into the blood, brain, and central nervous system [5].

There is a pressing need to assess the impact of cyanoHABs on neurodegenerative and other diseases due to their global and widespread occurrence, their production of toxins, and the multiple possible exposure routes [20]. Furthermore, a small set of studies provide nascent linkages between cyanoHAB exposure and neurodegenerative disease, possibly a result of proteinopathy [21], receptor activation [22], or oxidative stress [23], although the mechanisms remain unclear. To help address this need, the present study developed and evaluated life course residence-based exposure measures for cyanobacteria toxins using geospatial estimates of cyanoHAB that provide high temporal and spatial resolution suitable for application in large-scale environmental epidemiological studies. Using these measures, we investigated the effects of potential cyanoHAB exposure on the survival of amyotrophic lateral sclerosis (ALS), a progressive and fatal neurodegenerative disease previously linked to environmental exposures [24,25]. Our findings provide insight into the nature of geospatial-based cyanobacteria exposure metrics and raise important questions about their impact on the survival of this devastating disease.

## 2. Materials and Methods

### 2.1. Participants

CyanoHAB exposure metrics were developed for participants in a single-center, prospective observational study of ALS. Participant recruitment details were previously published [26,27,28,29,30,31]; these studies also discussed environmental, occupational, and genetic risk factors in this population. In brief, all patients meeting ALS Gold Coast criteria [32] were recruited during clinical visits to the University of Michigan Pranger ALS Clinic. The sole eligibility criterion for patients was the ability to provide informed consent in English. Participants were asked to complete a comprehensive exposure assessment—the MI-STORY survey (Michigan lifestyle Information, Sports and head Trauma history, Occupations, and Residences since Youth) [27,28,29,30,31,33]—which collects detailed information on demographics, military service history, hobbies, and home characteristics. All participants provided informed consent. The study was approved by the University of Michigan Institutional Review Board (IRBMED, HUM28826, HUM148060).

### 2.2. Residential History and Geocoding

To develop a lifetime residence history, participants were asked to list their home following birth (and birth date) and all prior residential addresses 30 years prior to enrollment, along with the move-in and move-out month and year for each residence or, alternately, their age when move-in or move-out occurred. These data were extensively reviewed, cleaned, and processed to identify and correct gaps, misspellings, overlaps, and other anomalies. In many cases, we were able to impute missing dates based on the available data, i.e., dates of the former and following residence. We also attempted to obtain missing data by following up with participants and reviewing clinical records.

Geocoding used a multistep procedure involving cleaning and reviewing the data, initial and final geocoding, and imputation for addresses that could be localized. Final geocoordinates were required to be confirmed by at least two geocoding engines. In the relatively few cases (<8% of addresses) where a 1 km localization could not be assured, e.g., a missing house number on a long street, we used other information (e.g., ZIP Codes) to constrain the location, estimate cyanoHAB exposure at multiple residential locations along the street or area, and then averaged these values.

### 2.3. CyanoHAB Exposure Estimates

Four scenarios may portray most exposure to cyanoHAB-related toxins. First, if the drinking water source is contaminated and water treatment is inadequate, exposure can occur via ingestion and inhalation, e.g., during showering and other domestic water use [16]. Large-scale examples of this scenario include the cyanoHABs occurring in western Lake Erie, most dramatically in 2014, and along the Maumee and Ohio Rivers in 2022, for which well-publicized water advisories were issued [34,35]. Additionally, exposures might occur around inland lakes, such as when individuals use shallow or leaky private wells. Second, for individuals who swim, fish, boat, water ski, or recreate in a contaminated water body, exposure may occur via skin contact, ingestion of water, and spray aerosolization with subsequent inhalation [9,11,17]. Authorities monitoring water bodies typically restrict beach access and provide warnings when algal blooms or, more commonly, *E. coli*, exceed guideline values, which might reduce the most severe exposures. Third, for individuals located near or downwind of the water body, waves, chop, surf, boating, water skiing, and other activities can aerosolize contaminated water, which can travel downwind and result in environmental inhalation exposure [11,19]. Fourth, and well documented in the literature, consumption of contaminated seafood can result in acute exposures [16,18]. The likelihood of exposure and the dose in each scenario depend on many factors, including the frequency and duration of events, breathing and ingestion rates, and environmental factors influencing the concentration and dispersal of toxins [5,36,37].

Exposure estimates for cyanoHAB toxins developed in this study were based on life course residential proximity to HABs detected and quantified using remote sensing data of cyanobacterial cell counts, which provide wide spatial coverage and nearly two decades of data. While satellite data can provide robust estimates of cyanobacteria cell counts, [38], the derived exposures are indirect and approximate for many reasons: actual exposure routes are unknown; types and levels of cyanoHAB toxins are rarely determined in water, air, biofluids or other media; toxins may not be present in blooms; and numerous factors affect the likelihood and magnitude of exposures even if toxins are present. Recognizing the surrogate nature and uncertainty of the exposure estimates, we utilized multiple measures that were complemented with several sensitivity and confirmatory analyses.

The proximity specification for the exposure metric, operationalized as a circular buffer around a residence, depended on the exposure pathway. Considering domestic water ingestion and use, public or private water providers serving large residential populations may utilize surface water sources that can be prone to cyanoHAB-related contamination, especially since typical filtration practices do not fully remove cyanobacteria toxins. In rural areas, the water source for a residence is often a private well. Water from deep wells normally would not be contaminated by cyanoHABs or other contaminants present in surface waters. However, residences with shallow wells have potential for exposure, particularly since few residential systems have filters that remove cyanobacteria toxins, and the risk increases with older wells and leaky casings that can draw in contaminated water. These factors suggest that residential proximity to cyanoHAB events would be a risk factor, particularly for individuals with private wells living close to contaminated water bodies. Ingestion exposure also can occur from incidental contact during swimming and other recreational activities. For individuals living at the lakeside, little travel might be required for such exposure. However, boating (or other transport) might take them short distances from their residence (perhaps 1–5 km), e.g., to visit a favorite beach. For individuals living elsewhere and traveling by road, distances traveled for water recreation might be less constrained, but again, nearby locations would likely be favored; thus, distances up to 10 km might be reasonable. In all of these scenarios, authorities detecting HAB outbreaks would subsequently issue drinking water and surface contact advisories. However, this largely applies to only larger, more severe, and prolonged events, relatively few of which were detected. Considering inhalation, exposure to aerosolized and airborne toxins will be highest near the source and decrease with distance due to dilution, deposition, and inactivation mechanisms. The atmospheric half-live of microcystin, for example, may be as short as 6 min during sunny summer conditions in the presence of ultraviolet (UV) radiation, ozone (O_3_) and other oxidants that promote inactivation, which corresponds to typical travel distances of ~1 km; however, half-lives at night (multiple hours) would permit much longer atmospheric transport [37,39] though other removal and dispersal mechanisms would tend to lower concentrations. Overall, no single buffer size accounts for all exposure pathways, the many factors influencing concentrations, and individual mobility. Consequently, we tested buffer radii ranging from 0.25 km, the smallest reasonable given the resolution of the datasets, to 10 km, a relatively long distance for water and airborne transport (although not necessarily long for personal mobility). As shown below, a 10 km buffer likely gave many false positives and reduced exposure contrasts.

Data from the Cyanobacteria Assessment Network [40], which uses optical imagery obtained from the Medium Resolution Imaging Spectrometer onboard Envisat (MERIS; 2002–2012) and the Ocean Colour Land Imager onboard Sentinel-3A (OLCI; 2017–present) and Sentinel-3B (2018–present), were downloaded from https://oceancolor.gsfc.nasa.gov/about/projects/cyan/ (accessed on 10 November 2024). The cyanobacteria index (DN), which provides daily to weekly estimates of cyanobacteria concentrations within 300 × 300 m pixels for water bodies in the contiguous US states and Alaska, quantifies cyanobacteria levels in 2192 satellite-resolvable lakes in the conterminous USA [41]. Statistics were developed from the 7- and 14-day maximum files available, excluding index values 0, 254, and 255 (representing below the limit of detection threshold, land pixels, and no data, respectively). The index value was converted to cyanobacteria abundance (CI) using the formula CI = 10^(DN×0.011714−4.1870866)^, which gave abundances from 0 to ~7,000,000 cells/mL. Both annual average and annual maxima were calculated across the 7- or 14-day files. While 2002–2023 data were available, 2012–2015 data had significant time gaps, particularly in summer when blooms often occur. To obtain a complete record, data for these years were interpolated using 2010 through 2017 data, and 2002–2003 data were extrapolated to provide estimates for 1995–2001. Results were insensitive to these approximations, as indicated by correlation and scatterplots of exposure metrics, a result of the exposure time window duration being at least 5 years, the few residential time histories that fell mainly or exclusively into the missing data period, and the temporal correlation in exposure (discussed below). As described below, the complete dataset was used to develop a set of concentration metrics for cyanobacteria exposure that differed in buffer size, cyanobacteria concentration metric, and exposure window, representing different assumptions regarding the likelihood and route of exposure.

**Figure 1:**
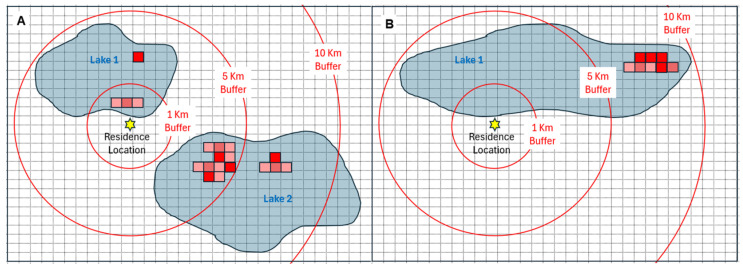
Hypothetical exposure scenarios showing the effect of buffer size. (**A**) CyanoHAB exposure captured in 1, 5, and 10 km buffers (red line). (**B**) CyanoHAB exposure captured only in a 10 km buffer. The grid depicts a 300 m grid resolution of satellite pixels. Portions of 1, 5, and 10 km buffers around a residence are shown as red partial and complete circles. Cyanobacteria levels are shown as colored pixels from low (pink) to high (red) concentration.

Buffers centered on each residence with radii of 0.25, 0.5, 1, 2.5, 5, and 10 km were considered (Figure 1). Small buffers represent cyanoHABs occurring very close (e.g., within 0.25 km) to the residence; larger buffers (e.g., 10 km) tend to include more lakes and more cyanoHAB events. As noted earlier, the wide range of radii reflects different exposure pathways (e.g., inhalation and ingestion), uncertainties associated with an individual’s mobility (e.g., travel for swimming or boating in lakes), and variation in cyanoHAB transport (e.g., dispersal of windblown aerosols). The water area in each buffer was determined using the “zonal statistics as a table” function in ArcGIS Pro 3.3.0. As depicted in Figure 1A, the number of lakes and the surface area of cyanoHAB-contaminated water will increase with buffer radius. In Figure 1B, the same lake was included in the three buffers, but cyanoHAB-contaminated water was included in the 10 km buffer only. A recreational boater or swimmer living in a lakeshore residence (Figure 1B) might be exposed since the bloom occurs in the same lake, though this would not be captured with the 1 or 5 km buffer, leading to a false negative. Trade-offs and factors affecting exposure related to buffer size are discussed later. To inform the derivation of and tune the models for cyanoHAB exposure metrics, residential data from controls in a retrospective case/control study of ALS were utilized [26,27,28,29,30,31].

**Figure 2:**
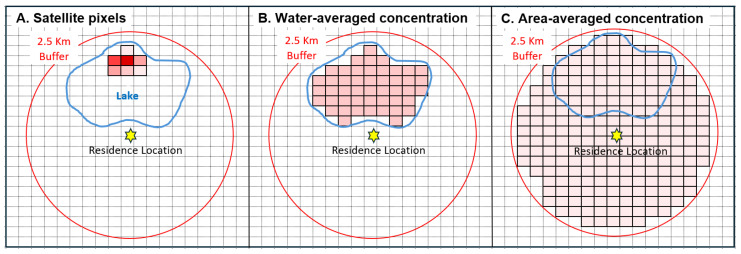
Comparison of three concentration metrics for a hypothetical lake (blue line), 2.5 km buffer (red line), and 300 m grid (satellite pixels). (**A**) Depiction of pixels with HAB detections representing the highest daily or annual average levels in a year. (**B**) Equivalent water-averaged concentration metric. (**C**) Equivalent land-averaged concentration. Cyanobacteria levels are shown as pixels colored from low (pink) to high (red) concentration.

Four concentration metrics were considered. The first, the annual daily maximum concentration, is the single highest daily cyanobacteria concentration occurring in any buffer pixel, extracted using the zonal statistics function. This provides a worst-case condition, e.g., inhalation and/or ingestion exposure for the worst bloom that year in the buffer (Figure 2A). The second, the water-averaged concentration, is the annual average cyanobacteria concentration in water bodies in the buffer, derived by averaging concentrations across the water area. Depicted in Figure 2B, the average concentration in the water is assumed to reflect long-term exposure. This metric may be relevant for water contact and possibly drinking water exposure. However, it has the disadvantage that the water body could be small, distant from the residence with a large buffer (e.g., 10 km), and not visited, thus yielding a false positive. The third metric, the area-averaged cyanobacteria concentration, is the water-averaged concentration adjusted by the ratio of water/land area in the buffer, providing a more representative estimate of long-term exposure and possibly relevant for the airborne exposure route (Figure 2C). In the example just discussed, results would be downweighted. The area-averaged value would be high if both the water-averaged cyanobacteria concentration was high and water bodies constituted much of the buffer area. Finally, the fourth metric used the annual daily maximum cyanobacteria concentration averaged across water areas in the buffer, potentially increasing the spatial representativeness of the worst-case metric. Each exposure metric was calculated annually for the 1995–2023 period. Numerous factors influence the accuracy of the cyanoHAB data themselves as well as the likelihood of exposure to cyanoHAB toxins via inhalation and ingestion pathways (further discussed below). Thus, these metrics are considered relatively crude but useful surrogates that can be derived from the available data.

The residence duration-weighted concentration average was determined for each individual using the residences occupied in each exposure window. Nine exposure windows were considered to potentially identify periods that might account for the disease latency and accumulated exposure, specifically, 15–20, 10–15, 5–10, 0–5, 10–20, 0–10, and 0–20 years before symptom onset; the period after onset to death (called “0–5 years after”); and the cumulative total (20 years before onset to death). In all cases, the average exposure at each residence location was weighted by the duration that the residence was occupied in the exposure window.

The combination of six buffer sizes, four exposure metrics, and nine exposure windows yielded 135 different measures of cyanoHAB exposure. Only selected measures were evaluated for ALS survival based on rates of cyanoHAB detections, similarity between measures, and the other analyses reported below.

### 2.4. Survival Analysis

Survival was based on date of diagnosis and evaluated using Cox proportional hazards models adjusted for age at diagnosis, sex, bulbar vs. non-bulbar onset, diagnostic El Escorial criteria, revised ALS functional rating scale (ALSFRS-R), and time between symptom onset and diagnosis, following our published studies [27,29,30,31,33,42,43]. Models were fit for post-diagnosis survival with subsequent Benjamini–Hochberg correction to control the false discovery rate for each exposure window. These models used the log of the cyanoHAB exposure. Models using untransformed cyanoHAB scores were also run, as were models that were not adjusted for covariates. Similar Cox models were used to assess potential interactions between cyanoHAB scores and factors potentially related to cyanoHAB exposure, specifically the residential water source (private well or city supply), participation in fishing and/or swimming, and participation in hunting. The covariates described above were used with the model Y ~ X β_1_ + **Z γ** for individuals reporting fishing/swimming and Y ~ X β_0_ + **Z γ** for individuals not reporting fishing/swimming where X = cyanoHAB measure, β_1_ = estimated hazard ratio for fishing/swimming, β_0_ = estimated hazard ratio for not fishing/swimming, **Z** = adjustment covariates and **γ** = estimated coefficients. The same model was used for the water source, with this variable replacing fishing/swimming. As a further sensitivity analysis, the dataset was stratified by these variables, and models were re-estimated. The interactions and stratified analyses excluded individuals who did not provide responses to questions about water source, fishing/swimming, and hunting activities, which slightly reduced the sample size. Covariate-adjusted survival curves were developed using Cox, Kaplan–Meier, and Royston–Parmar plots, the latter two sets using 10 iterations, for select cyanoHAB exposure metrics and two equally sized groups, as well as quartiles. Differences in median survival were calculated.

## 3. Results

### 3.1. Residence History and Geocoding

**Figure 3:**
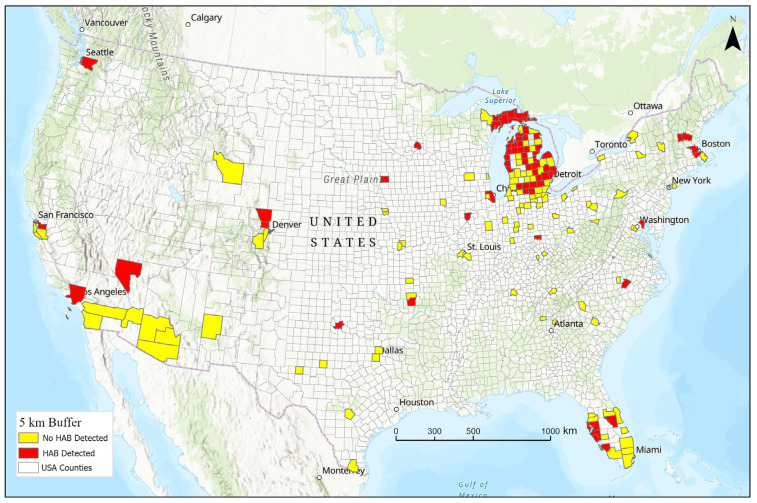
Map showing the counties of study participants’ residences. Includes 1504 residences in 178 counties, of which 69 counties had cyanoHAB occurrences during the lifetime exposure window. Red color depicts residences within 5 km of a cyanoHAB event when the participant lived there; yellow depicts counties without cyanoHAB events when the participant lived there.

A total of 4498 residences were reported for study participants. Of these, 3380 addresses were complete and successfully geocoded, and another 632 addresses had partial information that allowed geocoding but with less precision (typically with 2–3 km accuracy). The duration each residence was occupied was determined for each exposure window. A total of 1582 residences were occupied during the exposure windows considered (20 years prior to onset to up to 5 years after onset) of the 4012 residence locations geocoded. Residences were located mostly in Michigan, but also in Florida, California, other states, and other countries. The analysis excluded foreign locations, which represented only a small fraction of residences considered (N = 121, 3.1%). As seen in Figure 3, many cyanoHAB events occurred near study participants’ homes, e.g., within 5 km.

### 3.2. Cyanobacteria Exposure Estimates at the Residence-Level

This section explores the effects of buffer size, concentration metric, and year on cyanobacteria exposure estimates at 3380 residences with complete and precise locations. Exposure windows were not considered to avoid the effects of residence history. Participant-level life course exposure estimates are examined later.

Table 1 lists descriptive statistics for six buffer sizes and four cyanoHAB concentration metrics (water-averaged, area-averaged, annual daily maximum concentration, water-averaged annual daily maximum concentration). Cyanobacteria concentrations at the satellite (pixel) level spanned a wide range (0 to 7 × 10^6^ cells/mL). The residence-based estimates reflected this range but also depended on buffer size and concentration metric. Table 2 shows Spearman-rank correlation coefficients for six buffer sizes and four concentration metrics for the year 2019. (Supplementary Materials Table S1 shows that results for 2005 were similar.) Correlations significantly dropped between buffer sizes, and exposures in the smallest and largest buffers had low correlation (R ≤ 0.1), again highlighting sensitivity to buffer size. Within a given buffer size, however, the four metrics were highly correlated for smaller buffers (≤5 km radius). As discussed later, alternate concentration, metrics that might better represent exposure could be developed with additional information, e.g., locating the city water sources vis-à-vis HAB locations, or matching the timing of water recreation activities with cyanoHAB events. Still, the correlation between the concentration metrics suggests that differences between peak and average cyanoHAB did not substantially affect epidemiological models (e.g., survival models discussed later) or other analyses that compared relative differences in exposure.

As noted, results were sensitive to buffer size. Small buffers rarely had cyanoHAB events (any concentration > 0 cell/mL), and zero concentrations were estimated for 99.4, 97.3, and 93.5% of residence locations for the 0.25, 0.5, and 1 km radii buffers, respectively. Such small buffers were unlikely to contain water bodies, especially since a pixel representing a water body (300 m × 300 m) must not contain any land area for cyanoHAB extraction. This can be particularly limiting for shoreline residences, and it increased the likelihood of false negatives. For these reasons, as well as errors in positional accuracy (described later), buffers below ~1 km appeared too small to provide meaningful results. In contrast, most (59.4%) residences in the 10 km buffer experienced cyanoHAB events (40.6% had zero concentrations). However, large buffers have limited plausibility for representing actual exposure pathways, and they reduce exposure contrast and the ability to distinguish exposed and unexposed individuals since everyone in the buffer has the same calculated cyanoHAB exposure. Thus, large buffers will yield many false positives. Overall, buffer size should balance the likelihood and plausibility of exposure, the ability to discern exposure contrasts, and the spatial resolution of the available datasets. In the present study, intermediate-sized buffers using 2.5 and 5 km radii appeared to best reconcile these competing factors, and these radii gave cyanoHAB detections at 17 and 34% of the residence locations, respectively.

Cyanobacteria concentrations, excluding non-detects, had approximately a log-normal distribution. As an example, log probability plots for the water-averaged concentration showed a log-normal distribution for much of the data (straight lines on the plot), but the left hand tail was large and truncated at zero, especially for small buffers, while the right hand tails (highest values) tended to be limited to a maximum value (Supplementary Materials Figure S1). Larger buffers shifted the distribution to the right (higher values), again showing the sensitivity to this parameter. However, the shifts did not necessarily apply to the highest values of the water-averaged concentration metric. For the water-averaged concentration, this reflected the inclusion of additional pixels in larger lakes and the likelihood that only a subset of the lake experienced very high cyanoHAB concentrations. Thus, there was a countervailing effect with buffer size, but this affected only the highest concentrations. Buffer and lake size—and many other factors—can influence exposure, e.g., large lakes (especially if oriented along prevailing winds) might more frequently experience wave chop resulting in aerosolization [9,37,44], and large lakes might have more beaches, water skiing, and be visited more frequently.

The WHO guidelines, which are intended to be protective for low and moderate health risks associated with direct contact with cyanobacteria-contaminated water (20,000 and 100,000 cells/mL, respectively), were frequently exceeded for the maximum concentration metric and the larger buffers; these guidelines also were exceeded at 6.1 and 1.3% of residences with a 5 km buffer (Table 1). The guidelines were much less likely to be exceeded for the water- and area-averaged metrics. (Table 1 and Figure S1 use multiyear averages that smooth out annual fluctuations.)

The general long-term increase in cyanoHAB frequency and concentration that occurred over the past decades due to climatic shifts and other environmental factors was reflected in cyanobacteria concentration probability plots (Figure S2). While year-to-year variation was considerable, levels from 2002 to 2011 were fairly similar: considering the water-averaged metric and a 5 km buffer shown in Figure S2, an average of 1.1% of residences exceeded the lower WHO guideline and cyanoHAB events occurred near 18.3% of residence locations (any non-zero level within 5 km). From 2016 to 2023, 1.6% of residences exceeded the guideline level, and events occurred near 54.8% of residences. Similar results were seen for the other concentration metrics. Thus, the geospatial data reflect both higher concentrations and increased prevalence of cyanobacteria blooms in recent years. Changes in satellite platforms, data processing protocols, and data gaps also may have affected estimates of temporal variability.

### 3.3. Life Course Cyanobacteria Exposure Estimates: Individual-Level Data

The residence-level analysis just discussed excluded effects due to an individual’s residence history and a specific exposure window. Table 3 and Table 4 provide descriptive statistics and correlations for cyanoHAB exposure at the individual level for six exposure windows (0–20, 10–20, 0–10, 0–5 years prior diagnosis, 0–5 years after diagnosis, and lifetime), the four concentration metrics, and the 5 km buffer, selected as a buffer size that balances false negatives and false positives (as noted earlier). (Table S2 gives results for all buffer sizes as well as additional statistics.) Within each concentration metric, higher exposures tended to be associated with longer and more recent exposure windows. Correlations were highest among metrics using the same exposure window, and lowest for long time gaps between the windows (e.g., between 10 and 20 years prior to onset and 0 to 5 years after). Compared to residence-level statistics (Table 1), individual-level statistics showed higher cyanoHAB exposure, e.g., percentages of individuals living near (any) cyanoHAB event for lifetime exposure window increased to 3.4, 12.1, 24.2, 64.6 and 91.3%, for the 0.5, 1, 2.5, 5 and 10 km buffers, respectively (Figure S3), and a larger fraction experienced levels over the WHO guideline, e.g., 48.8% for the 5 km buffer, lifetime exposure window, and maximum concentration metric. The increased likelihood and exposure level at the individual level (particularly for long and recent exposure windows) occurred since most individuals occupied several residences, residences can change to locations near water bodies, and cyanoHAB frequency and intensity have tended to increase.

### 3.4. HAB Exposure and ALS Survival

This section examines survival rates for ALS cases. Selected demographic variables, including those associated with ALS survival in prior studies, are provided in Supplementary Materials Table S3. The median participant age was 64 years at diagnosis (N = 309), 57% were male, 98% were white, 13% had prior military service, and 72% had at least some post-secondary education. A median of 1.04 years elapsed between symptom onset and diagnosis, and 2.32 years between diagnosis and death.

Survival models for cyanoHAB exposure showed significant associations (*p* < 0.05, Q < 0.05) for the 2.5 and 5 km buffers, all four concentration metrics, and the four exposure windows prior to onset (Table 5). Hazard ratios (HRs) in these models mostly fell between 1.10 and 1.20. Generally, the exposure windows and the four concentration metrics yielded similar results, as did unadjusted models (Table S4). Overall, the 0–10 and 10–20-year exposure windows using the 5 km buffer attained the largest and most significant HRs. It was unsurprising that the small buffers (≤1 km) did not yield statistically significant results since they had few participants with cyanoHAB exposures (Table 1). The 10 km buffer also did not yield significant results, likely reflecting higher exposure measurement error as noted previously.

**Figure 4:**
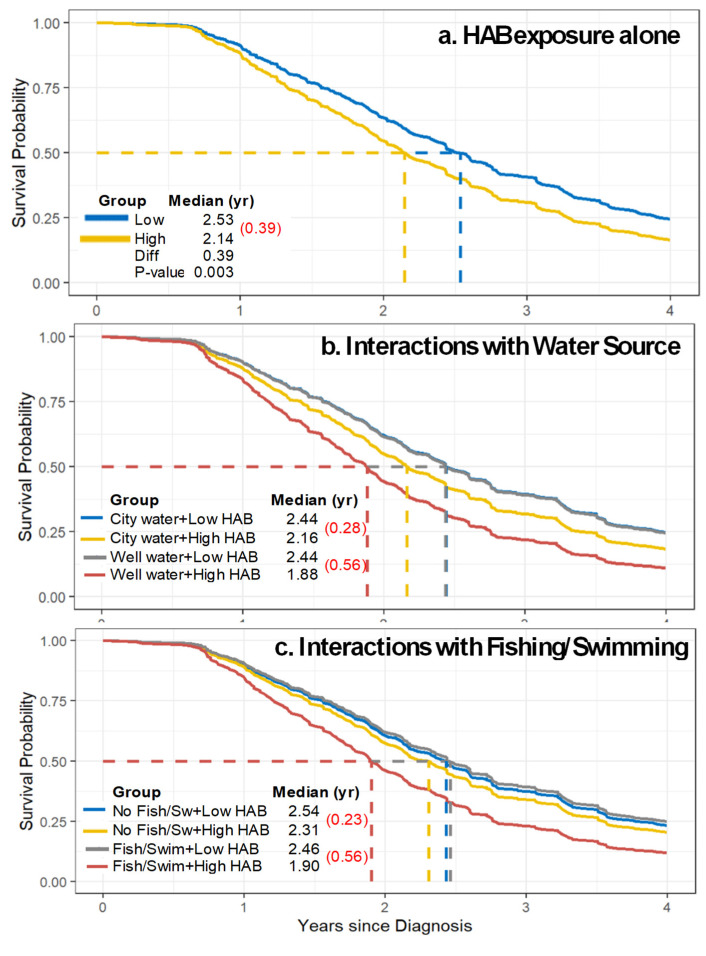
Covariate-adjusted Cox survival curves for upper versus lower exposure groups for the water-averaged concentration metric with a 5 km buffer and a 0–20-year exposure window. (**a**) CyanoHAB exposure alone; (**b**) Interaction with water source; (**c**) Interaction with fishing/swimming activities. Median survival is shown for each exposure group (and interaction). Survival differences in parentheses and red. In 4b, lines for “City water + Low HAB” and “Well water + Low HAB” overlap. N = 303.

Cox survival curves using the 5 km buffer, water-averaged concentration, 0–20-year exposure window, and binary grouping of exposures showed a median survival of 2.53 years for the less exposed group versus 2.14 years for the more exposed group, equivalent to a 0.39 year survival difference (Figure 4a). Survival curves for the three other concentration metrics generated slightly larger differences for cyanoHAB exposure, shortening survival by 0.44 to 0.49 years (Figure S4). Kaplan–Meier and smoothed Royston–Parmar survival curves were similar (Figure S5). Cox survival curves computed using exposure quartiles had similar but longer survival for the lower two exposure quartiles (2.82 years), and shorter survival for the third and fourth quartiles (2.02 and 2.13 years in the fourth quartile, depending on metric), translating to 0.44 to 0.56 years shorter survival with cyanoHAB exposure (Figure S6). Survival curves for the 2.5 km buffer were similar. The similarity between the first and second quartiles reflects that little or no exposure occurred for a sizable fraction of individuals.

#### 3.4.1. Water Source

In the ALS cohort, 41% (97 of 239 reporting this information) of participants obtained drinking water from a private well. No difference by sex was seen (*p* = 0.4, Chi-squared test). Potential effects on ALS survival due to cyanoHAB contamination in the water source were investigated using interaction analyses with different buffer sizes, concentration metrics, and exposure windows. Table 6 shows results for the 5 km buffer; results for the 2.5 km buffer were similar. Statistically significant interactions (*p* < 0.05) were detected for having a private well and cyanoHAB in all exposure windows, with HRs that ranged from 1.24 to 1.44, which are higher than the HRs without interactions. The 0–20-year exposure window also had significant results after correcting for multiple comparisons (Q ≤ 0.05). In contrast, only two interactions with city water supply achieved significance (*p* ≤ 0.05), both with the 10–20-year exposure window, but not after the multiple comparison corrections. As a sensitivity analysis, survival models stratified by water source were tested, which again showed significant effects for residences with private wells for nearly all exposure windows, while effects for residences using city water were rarely significant (Table S5). The interactions and stratified models yielded similar findings, though the latter tended to have slightly larger HRs, mostly in the range of 1.3 to 1.6, with a private well. Again, results for the 5 and 2.5 km buffers were similar. Survival curves demonstrating interactions with water source (Figure 4b) suggest that roughly half of the difference in survival was due to the interaction with private wells. Survival curves for all four concentration metrics (Figure S7) showed that median survival with cyanoHAB exposure and private wells decreased by 0.56 to 0.73 years. The area-averaged concentration metric had the largest survival decrement. To an extent, this finding was consistent with the formulation of the area-averaged metric, which emphasized the likelihood of contact with contaminated water (or aerosols) across the buffer area. However, since this is not a positive confirmation and other metrics have shown similar results, this finding does not provide evidence of a preferred concentration metric for the residential water consumption and use pathway. Overall, the evidence was strong that shorter survival for ALS individuals living within 5 km of cyanoHAB events is, in part, driven by private well use. In contrast, a household without a private well utilized city (municipal) water and thus was likely to be at low risk of cyanotoxin contamination given the different and potentially distant water source, and potentially the additional water treatment and monitoring conducted by the water provider.

#### 3.4.2. Fishing/Swimming and Other Personal Activities

The participant survey queried several personal activities that might have increased exposure to cyanoHAB-related toxins. Among participants with ALS, males were more likely to fish and/or swim than females (72% versus 45%, *p* < 0.001, N = 248). Significant interactions (*p* ≤ 0.05 and Q ≤ 0.05) were found for fishing/swimming and cyanoHAB-related exposure on survival for all four concentration metrics and four exposure windows with the 5 km buffer, with one exception that was marginally significant (Q = 0.06 for the area-averaged metric with 10–20 year window; Table 7). HRs were mostly in the 1.30-to-1.38 range, higher than that seen in analyses without interactions. Importantly, no significant interactions were seen for individuals who did not report fishing or swimming. The stratified analysis, again used as a sensitivity analysis, generated similar results (Table S6). Model results for interactions and stratified analyses for fishing and swimming for the 2.5 and 5 km buffers were very similar, and the 1 km buffer also obtained statistically significant *p*- and Q-values for the 0–20-year exposure window among those reporting fishing and swimming, and no statistically significant results for those not reporting these activities. Survival curves utilizing interactions for fishing/swimming (Figure 4c) showed that the cyanoHAB exposure + fishing/swimming interaction shortened survival by 0.56 years, while the effect of cyanoHAB exposure without fishing/swimming was much smaller (0.23 years). Survival curves for the other concentration metrics showed larger effects (0.56–0.89 years; Figure S8), the longest was obtained for the area-averaged concentration metric (Figure S8A). Recognizing that the evidence is indirect, the likelihood of exposure due to fishing/swimming activities when an individual lived near a contaminated water bodies is reasonably high; said differently, an individual is less likely to have been exposed via fishing or swimming if cyanoHAB contamination was limited to small and distant lakes that were not visited. Overall, these strong and consistent results suggest significantly shortened survival for ALS patients who fished or swam in cyanoHAB-contaminated water bodies located within 5 km of their home, and they tend to support the area-averaged concentration metric.

In our survey, other variables that might portray personal activities associated with cyanoHAB exposure included hunting-related activities, including the use of guns, shooting, skeet, trap, or targets. Among participants with ALS, these activities were more likely among males than females (47% versus 3%, *p* < 0.001, N = 248). However, we did not find significant effects on survival for these activities.

## 4. Discussion

This study developed and evaluated several residence-based proximity and concentration measures of cyanoHAB exposure over a spatial and temporal scale that has not been previously assessed due to methodological limitations. In applying these measures to explore associations with the survival of ALS patients, we found that over half of the study participants lived within 5 km of cyanoHAB events during their lifetime. Exposure measures using 2.5 and 5 km radii around residences up to 20 years prior to disease onset appeared to best balance the likelihood and plausibility of exposure while still resolving exposure contrasts. These buffer sizes also had the most significant impact on ALS survival. For any specific buffer dimension, the four concentration metrics had high correlation but different scales, reflecting differences between water-averaged, land-averaged, and maximum statistics. Cyanobacteria concentrations derived from the satellite data were roughly lognormally distributed, and maximum levels sometimes exceeded the WHO guidelines for low and moderate risk. While not intended to reflect neurotoxicity, these exposures could be associated with acute health impacts.

The residence-based proximity measures presume that individuals living closer to water bodies with cyanobacteria blooms are more likely to be exposed to cyanoHAB toxins. Such individuals may be more likely to obtain domestic water from the water body, may be more likely to visit, fish, swim, and recreate at the water body, and may live close enough to breathe aerosols from the water body. The epidemiological findings showed shorter survival for ALS patients who lived within 2.5 and 5 km from cyanoHABs. The interaction and sensitivity analyses showed significant effects for individuals who utilized private wells for domestic water, strong evidence for the ingestion and water use exposure routes, and yet stronger evidence for individuals who fished and swam, supporting ingestion, aerosol inhalation, and/or dermal uptake from water sports and wave action. The fishing/swimming association might also support the ingestion of contaminated locally caught fish. However, little consumption of harvested shellfish, which can accumulate toxins, occurs in the Midwest, where most study participants lived.

### 4.1. Exposure Scenarios and Exposure Measurement Error

The likelihood of exposure and the dose from ingestion and inhalation of cyanoHAB toxins depend on many factors, including the frequency and duration of events, breathing and ingestion rates, and environmental factors influencing the concentration of toxins [5,36,37]. To represent possible exposure scenarios with available data, exposure metrics were developed as proximity measures using residential histories and satellite-derived cyanobacteria estimates. The metrics differed in terms of the area or buffer size around the residence (0.25 to 10 km radii), the concentration statistic (annual average or maximum for water and land area), and the exposure window. The water-averaged concentration might be both more representative (less prone to outliers) and better reflect the potential for water ingestion and aerosol inhalation for direct (swimming) and near-direct contact (boating, fishing) activities than maximum statistics. Averaging also helps account for the spatial coverage of the cyanoHAB and its duration. The area-averaged concentration, similar to the water-averaged but reduced by the proportion of water area in the buffer, was designed to reflect inhalation of environmental aerosols. While the metrics had similar HRs and significance, the area-averaged metric showed the greatest decrements in survival in the interactions analysis, namely, 0.72 years with well water and 0.89 years with fishing/swimming, possibly because this metric better accounts for the likelihood of near-shore residences and that nearby and contaminated water bodies will be visited. The maximum metrics represent worst-case conditions, e.g., the highest daily measurement, possibly relevant to inhalation and/or ingestion exposure from the worst-case bloom. Given satellite resolution, the smallest worst-case area was 9 ha, a sizable area but possibly one that many individuals might not encounter. Further, a 9 ha area may not be representative of a large lake. While indirect contact via lake water used domestically, e.g., at residences using shallow wells, was potentially captured by the four concentration metrics, particularly as an interaction with a private well, available data do not allow an assessment of the likelihood or representativeness of the exposure estimates. Further, some individuals likely received exposures by multiple routes.

Geospatial cyanoHAB data have the advantages of excellent spatial and temporal coverage but may not reflect individual and population exposures or risks in survival analyses like the present study and in other epidemiological applications due to exposure measurement error, which can yield biases, artifacts, false positives, and false negatives. While the spatial resolution of satellites continues to improve, many issues remain, e.g., small water bodies cannot be distinguished, pixels containing both water and land areas (coastlines) cannot be assessed, and cyanoHABs occurring in deep or turbid waters will be missed or under-reported [8,38,45]. In addition, satellite data do not directly measure cyanoHAB toxin concentrations, and relationships between cyanoHAB biomass and microcystin and other toxin concentrations are variable [45,46]. Aerosol concentrations and lifetimes can depend strongly on meteorological conditions and aerosolization mechanisms [9,37,44]. Further, each pixel (300 × 300 m) likely has large variations in toxin concentrations [41], and time-averaged estimates from satellite imagery may underestimate levels due to mixing of the water column, although using weekly composite images that preserve the pixel maximum may minimize this problem [41] (as used here in the maximum concentration metric). Blooms can be very transient and possibly not associated with toxins, much less exposure. Lastly, satellite data do not reflect exposure to cyanoHAB toxins occurring from terrestrial sources, e.g., water treatment plants in bloom-affected areas disposing of sludge in agricultural fields [47] and cooling tower aerosols [19]. Despite these limitations, geospatial data’s broad spatial and temporal coverage represents a potentially useful surrogate of exposure to very potent toxins, particularly when coupled to individual-level information on activities that can cause contact with cyanoHABs, e.g., participation in water sports and use of surface water.

More knowledge of cyanoHAB exposure routes would help refine geospatial exposure estimates. If the operative route was consumption and use of contaminated surface or groundwater, a preferred exposure surrogate may be the distance from the nearest edge of the contaminated water body to the residence location (or well), reflecting the likelihood of the drinking water source being contaminated. In this case, information regarding the water source of participants was essential, and while we knew whether a private well was used, information regarding well depth, age, and condition could improve reliability. If the exposure route is inhalation and incidental ingestion during water sports (including swimming and boating), additional information regarding the frequency, timing, and conduct of these activities could be helpful. Metrics for both exposure routes might utilize lake-wide concentration statistics, such as the water-averaged or maximum cyanobacteria concentration. If water sports are not at play but inhalation of environmental aerosolized toxins is suspected, then the area-averaged metric, possibly with a directional component to reflect prevailing winds and possibly adjusted for aerosol generation (e.g., using concurrent wind speed), atmospheric lifetime (ambient UV and O_3_ levels), and dispersion (turbulence) might be suitable. For vacation (secondary) homes, which are frequently near water bodies, an additional refinement might consider the intersection of occupancy patterns (e.g., weekends or summer periods) and HAB events.

The cyanoHAB exposure estimates dependence on buffer sizes, reflecting a concern for all proximity measures. While distance-based buffers can be protective and serve many applications [48], small buffers (~1 km) can yield errors due to limits in the positional accuracy of geocoding [49]. Moreover, the smallest buffer used here, 0.25 km, is smaller than the 300 m resolution of the geospatial data and included few cyanoHAB events, and thus was too small for the present application. In contrast, large buffers (e.g., ≥10 km) decreased exposure contrasts, yielded many false positives, and most importantly, appeared inconsistently and implausibly associated with frequent and high exposures. Importantly, any discrete buffer distance used to define a metric can introduce bias. To avoid sensitivity to buffer size, cumulative distributions of proximity, e.g., distance to event of concern, have been proposed [50]. Literature reports have focused on distance to discrete sites, such as waste sites, schools, and fire stations, rather than the presence, count, and intensity of features within a buffer, features pertinent to cyanoHABs. Thus, the cumulative distribution approach is not entirely satisfactory for life course exposure to cyanoHABs, particularly since blooms vary considerably in concentration and their spatial and temporal extent, effects that are incompletely captured by proximity. Further, when multiple residence locations are considered over the life course, the closest event may not reflect exposure or the exposure window. Distance-weighted and possibly directional (for airborne exposures) buffers might lessen the sensitivity to buffer size, as shown for traffic-related air pollution exposures [51].

There was sensitivity to the exposure window. The short window following symptom onset did not show meaningful results, suggesting that current addresses of ALS patients (e.g., upon diagnosis) are not informative of cyanoHAB exposure. Longer exposure windows prior to symptom onset provided significant and more stable results, possibly due to being more representative and including more cyanoHAB events. However, participant-provided information is subject to recall bias, particularly for details occurring long ago, e.g., old residence addresses and activities. Even if the analysis had revealed strong trends that favored a specific exposure metric, uncertainties of the proximity measures would preclude positive identification of a window of vulnerability, much less the exposure route. Ground-truthing using air, water, or biological measurements is required to select the most appropriate geospatial metric. With these caveats, our results suggest that exposure windows from 0 to 10 years or, ideally, 0 to 20 years prior to disease onset are preferable. Longer windows are not possible at present, given the advent of satellite data around 2002, but will be available for future ALS cases and would warrant evaluation. Importantly, the results suggest a minimum of 10 to 20 years of exposure information is desirable.

### 4.2. Plausibility of Survival Effects

ALS is known to be influenced by genetic and environmental factors [24,25], including exposure to organic compounds [28,42,52], metals [53,54], and occupational stressors [33]. Our study is the first population-level study of ALS survival using geostatistical metrics for exposure to cyanobacteria toxins, specifically, satellite-based estimates of cyanobacteria levels in water bodies near residences occupied in the last 20–25 years of life. Despite the likelihood of exposure measurement error, the cyanoHAB exposure metrics were associated with survival decrements following disease diagnosis, ranging up to 0.89 years, a significant fraction of the 2–3-year survival period typical for ALS. The interactions and stratified analyses accounting for domestic water supply and swimming and fishing activities strongly support the association with cyanobacteria toxins and diminish the likelihood of confounding by socio-economic factors, including occupational exposures and stressors noted above.

As noted, the role of cyanoHAB on ALS has focused on BMAA-induced toxicity. BMAA was initially linked to ALS when it was, in part, implicated as an exogenous exposure and cause of the Western Pacific ALS–Parkinsonism–Dementia Complex (ALS-PDC) [55], although inconsistencies are noted [56]. Of note, BMAA has been detected in brain [7,57] and CSF [58] samples from participants with ALS, supporting its access to the CNS. Moreover, when administered to *Chlorocebus pygerythrus*, BMAA led to a TDP-43 proteinopathy [21]. The leading BMAA toxicity hypothesis is that BMAA is misincorporated into proteins instead of serine, causing protein misfolding, although some dispute this [56]. Additional potential mechanisms of BMAA toxicity include AMPA/kainate receptor activation [22]. NMDA and mGluR5 receptor activation, and oxidative stress [23]. Alternatively, toxicity might be unrelated to BMAA and instead due to other toxins released from cyanoHABs such as microcystins [59], nodularin, or anatoxin-a [60]. Nonetheless, prior studies have associated cyanoHAB with ALS risk, while others have linked cyanoHAB-related toxins to ALS. A study of ALS patients in New Hampshire found higher ALS risk if living within 0.5 miles of a lake with a history of cyanobacterial blooms [59,61] potentially linked to adverse water quality [17], and increased likelihood of ALS clusters within 10 to 30 km buffers of areas with more compromised water quality [62]. A study in several regions of Italy showed a non-significant increased ALS risk for those living 100 m from a water body [63].

ALS cases have distinct geographic distributions. If exposure to cyanobacteria toxins is a meaningful risk factor, then the large number of inland lakes prone to cyanoHABs in the US Midwest region (e.g., Michigan, Wisconsin, Minnesota, Illinois and Indiana) might explain the regional patterns of disease incidence, which is much higher that other US regions [64]. However, other stressors are present in the region, including exposure to chemicals in the production industries and agriculture, as well as exposure to legacy chemicals like PCBs via Great Lakes fish. Additionally, HAB species and toxins may differ regionally. Given the complexity of the disease, epidemiological approaches encompassing a life course and a broad exposomic approach appear highly promising. Such studies also might provide insight regarding the risk factors associated with ALS onset, in addition to the effects on survival examined here.

### 4.3. Recommendations

Several steps could confirm our results and ensure reproducibility. Ground-truthing using air, water, or biological measurements would complement the proximity measures and, as mentioned, might help to select the most appropriate geospatial metric. Exposure estimates might be refined with additional water-related survey questions, e.g., the type and frequency of water-related activities. For the small group of participants who reported second (e.g., vacation) homes, knowing the dates when visited also could refine exposure estimates. While most study participants lived in the US Midwest, participants also lived on the east and west coasts of the US. With a larger sample size, conducting sensitivity analyses or stratifying by region, climate, and water source could be revealing. Study participants were largely white, and analyses should be conducted of populations with different demographics.

## 5. Conclusions

This study has demonstrated the development of life course exposure estimates of cyanoHAB toxins using residence-based proximity and concentration metrics and applied these estimates to a population study of ALS survival. The geospatial data provide relatively complete, temporally- and spatially resolved information of cyanoHAB levels in surface waters, which, when coupled with residence history, individual behavior, and water supply information, appear highly informative and broadly applicable for identifying exposed individuals. CyanoHAB exposure was associated with a significant reduction in ALS survival, and significant interactions were seen for individuals using private wells as the source of water supply, especially for individuals engaging in swimming and fishing. While the associations in this study were strong and consistent with cyanoHAB exposure, the complex nature of exposures, the indirect measures, and other limitations in the geospatial exposure metrics are recognized. Importantly, exposure to cyanobacteria toxins is a modifiable risk factor. Thus, by avoiding high-risk activities associated with this exposure, ALS survival (and possibly ALS risk) might be improved. This may apply to other diseases, given the known toxicity associated with cyanoHABs. Additionally, local and national authorities could improve cyanoHAB forecasts, warning systems could consider lowering advisory levels to be more protective (thus shielding individuals from exposure), and assessments of the effectiveness of advisories could be undertaken.

While recognizing that gaps and challenges remain in our understanding of life course exposures to cyanobacteria toxins and their impacts, our results identify a new and modifiable risk factor affecting ALS survival. There is an urgent need for research to confirm and refine cyanoHAB exposure metrics and these epidemiological findings. We also suggest that geospatial estimates of cyanoHAB exposure used in population studies have the potential to discover new relationships with neurodegenerative and other diseases.

## Figures and Tables

**Table 1:** Residence-level statistics for six buffer sizes (0.25, 0.5, 1, 2.5, 5, and 10 km radii) and four cyanoHAB concentration metrics (area-average, water-average, maximum daily, and average annual daily maximum). Shown are the annual averages for the 2002–2023 period, excluding 2012–2015. N = 3380.

	Average	Stan. Dev.	Zero	>Low Risk	>Mod Risk	Percentiles (10^6^ Cells/mL)				
	(10^6^ cells/mL)		(%)	(%)	(%)	P75	P90	P95	P99	P100
B0.25_Area_Ave	0.0001	0.0025	99.39	0.13	0.02	0.0000	0.0000	0.0000	0.0005	0.1211
B0.5_Area_Ave	0.0002	0.0029	97.48	0.37	0.02	0.0000	0.0000	0.0000	0.0047	0.0949
B1_Area_Ave	0.0004	0.0036	93.83	0.63	0.02	0.0000	0.0001	0.0005	0.0109	0.0810
B2.5_Area_Ave	0.0011	0.0115	83.76	0.67	0.13	0.0000	0.0003	0.0012	0.0282	0.4637
B5_Area_Ave	0.0005	0.0035	66.99	0.33	0.03	0.0001	0.0005	0.0020	0.0067	0.1192
B10_Area_Ave	0.0006	0.0039	42.84	0.50	0.04	0.0002	0.0009	0.0016	0.0119	0.1306
B0.25_Water_Ave	0.0002	0.0041	99.39	0.22	0.06	0.0000	0.0000	0.0000	0.0011	0.1465
B0.5_Water_Ave	0.0009	0.0094	97.48	0.92	0.28	0.0000	0.0000	0.0001	0.0186	0.2125
B1_Water_Ave	0.0013	0.0117	93.83	1.37	0.36	0.0000	0.0004	0.0023	0.0401	0.3472
B2.5_Water_Ave	0.0085	0.0695	83.76	3.66	1.03	0.0005	0.0039	0.0127	0.2050	2.0081
B5_Water_Ave	0.0054	0.0226	66.99	5.82	1.24	0.0019	0.0070	0.0299	0.0857	0.4763
B10_Water_Ave	0.0083	0.0243	42.84	10.11	1.29	0.0043	0.0269	0.0453	0.1035	0.4377
B0.25_Maximum	0.0032	0.0525	99.39	0.60	0.53	0.0000	0.0000	0.0000	0.0476	1.5662
B0.5_Maximum	0.0151	0.1226	97.48	2.46	2.24	0.0000	0.0000	0.0077	0.3948	2.4447
B1_Maximum	0.0357	0.1934	93.83	6.06	5.49	0.0000	0.0559	0.1661	0.9632	3.3671
B2.5_Maximum	0.1049	0.3242	83.81	16.00	14.95	0.0757	0.2402	0.5432	1.6454	4.0444
B5_Maximum	0.2551	0.5129	67.09	32.61	30.91	0.2399	0.7356	1.4140	2.1588	4.7256
B10_Maximum	0.5593	0.7499	42.93	56.59	54.50	0.7130	1.6134	2.0261	3.4288	5.2808
B0.25_Max_Ave	0.0032	0.0515	99.39	0.60	0.53	0.0000	0.0000	0.0000	0.0472	1.5137
B0.5_Max_Ave	0.0136	0.1116	97.48	2.45	2.20	0.0000	0.0000	0.0066	0.3375	2.2576
B1_Max_Ave	0.0271	0.1501	93.83	6.03	5.03	0.0000	0.0356	0.1271	0.7701	2.6581
B2.5_Max_Ave	0.0689	0.2274	83.81	15.89	12.94	0.0350	0.1509	0.3219	1.1767	2.9026
B5_Max_Ave	0.1472	0.3261	67.09	32.47	26.97	0.1289	0.3615	0.8725	1.4263	3.3109
B10_Max_Ave	0.2724	0.4221	42.93	56.37	47.58	0.2776	0.8804	1.0956	1.9229	3.9112

**Table 2:** Spearman correlation coefficients for cyanoHAB exposures at the residence level for six buffer sizes (0.25, 0.5, 1, 2.5, 5, and 10 km radii), four concentration metrics, and calendar year 2019. Color coded from 0 to 1.0. N = 3380.

	B0.25_Area_Ave	B0.25_Water_Ave	B0.25_Maximum	B0.25_Max_Ave	B0.5_Area_Ave	B0.5_Water_Ave	B0.5_Maximum	B0.5_Max_Ave	B1_Area_Ave	B1_Water_Ave	B1_Maximum	B1_Max_Ave	B2.5_Area_Ave	B2.5_Water_Ave	B2.5_Maximum	B2.5_Max_Ave	B5_Area_Ave	B5_Water_Ave	B5_Maximum	B5_Max_Ave	B10_Area_Ave	B10_Water_Ave	B10_Maximum	B10_Max_Ave
**B0.25_Area_Ave**	1.00																							
**B0.25_Water_Ave**	0.99	1.00																			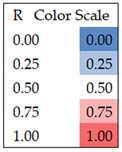			
**B0.25_Maximum**	0.92	0.95	1.00																					
**B0.25_Max_Ave**	0.92	0.95	1.00	1.00																				
**B0.5_Area_Ave**	0.52	0.51	0.48	0.48	1.00																			
**B0.5_Water_Ave**	0.46	0.47	0.44	0.44	0.98	1.00																		
**B0.5_Maximum**	0.44	0.45	0.46	0.46	0.95	0.96	1.00																	
**B0.5_Max_Ave**	0.43	0.44	0.46	0.46	0.94	0.96	0.99	1.00																
**B1_Area_Ave**	0.37	0.36	0.34	0.34	0.73	0.73	0.70	0.69	1.00															
**B1_Water_Ave**	0.33	0.33	0.30	0.30	0.69	0.69	0.66	0.66	0.98	1.00														
**B1_Maximum**	0.31	0.31	0.31	0.31	0.66	0.66	0.68	0.67	0.94	0.95	1.00													
**B1_Max_Ave**	0.29	0.30	0.30	0.30	0.61	0.62	0.64	0.64	0.90	0.93	0.98	1.00												
**B2.5_Area_Ave**	0.24	0.23	0.21	0.21	0.47	0.46	0.44	0.44	0.70	0.68	0.66	0.64	1.00											
**B2.5_Water_Ave**	0.20	0.20	0.19	0.19	0.40	0.41	0.40	0.40	0.58	0.60	0.58	0.58	0.90	1.00										
**B2.5_Maximum**	0.20	0.21	0.20	0.20	0.40	0.40	0.40	0.40	0.60	0.60	0.61	0.59	0.91	0.92	1.00									
**B2.5_Max_Ave**	0.17	0.17	0.17	0.17	0.35	0.35	0.36	0.36	0.51	0.53	0.54	0.55	0.81	0.93	0.94	1.00								
**B5_Area_Ave**	0.16	0.15	0.14	0.14	0.32	0.32	0.31	0.31	0.48	0.46	0.45	0.43	0.74	0.68	0.70	0.64	1.00							
**B5_Water_Ave**	0.14	0.14	0.13	0.13	0.29	0.28	0.28	0.28	0.40	0.41	0.39	0.39	0.61	0.66	0.62	0.62	0.92	1.00						
**B5_Maximum**	0.14	0.14	0.13	0.13	0.28	0.28	0.28	0.28	0.41	0.41	0.41	0.41	0.65	0.65	0.68	0.65	0.92	0.92	1.00					
**B5_Max_Ave**	0.11	0.11	0.11	0.11	0.22	0.22	0.23	0.23	0.30	0.32	0.33	0.34	0.46	0.57	0.55	0.61	0.75	0.88	0.89	1.00				
**B10_Area_Ave**	0.10	0.10	0.09	0.09	0.23	0.23	0.23	0.22	0.34	0.33	0.32	0.30	0.52	0.46	0.48	0.43	0.70	0.63	0.65	0.51	1.00			
**B10_Water_Ave**	0.09	0.09	0.08	0.08	0.18	0.18	0.18	0.18	0.23	0.24	0.23	0.23	0.33	0.37	0.35	0.36	0.49	0.56	0.52	0.51	0.85	1.00		
**B10_Maximum**	0.08	0.08	0.08	0.08	0.19	0.19	0.19	0.19	0.26	0.26	0.26	0.25	0.39	0.39	0.42	0.40	0.55	0.54	0.59	0.52	0.88	0.87	1.00	
**B10_Max_Ave**	0.05	0.05	0.06	0.05	0.11	0.11	0.12	0.12	0.12	0.14	0.15	0.16	0.14	0.25	0.23	0.29	0.26	0.39	0.39	0.52	0.54	0.80	0.75	1.00

**Table 3:** Individual-level statistics for the water-averaged concentration metric, four exposure windows, and the 5 km buffer. N = 584 individuals. Concentration in 10^6^ cells/mL.

Exposure Metric	Average	Stan. Dev.	Zero (%)	% > Low Risk	% > Mod Risk	P75	P90	P95	P99	P100
Water_Ave_0-20YrBefore	0.0040	0.0136	33.28	5.69	0.17	0.0012	0.0096	0.0227	0.0637	0.1983
Area_Ave_0-20YrBefore	0.0003	0.0012	33.28	0.17	0.00	0.0001	0.0006	0.0011	0.0043	0.0201
Maximum_0-20YrBefore	0.1777	0.3332	33.45	57.41	39.14	0.1876	0.5145	0.8426	1.7030	2.6558
Max_Ave_0-20YrBefore	0.1094	0.2240	33.45	53.45	25.00	0.0999	0.3165	0.5248	1.1233	2.4309
Water_Ave_10-20YrBefore	0.0032	0.0152	64.67	4.92	0.35	0.0001	0.0019	0.0156	0.0694	0.2295
Area_Ave_10-20YrBefore	0.0002	0.0008	64.67	0.00	0.00	0.0000	0.0002	0.0013	0.0036	0.0117
Maximum_10-20YrBefore	0.1140	0.3246	64.50	29.88	17.75	0.0579	0.3260	0.8115	1.6544	3.2858
Max_Ave_10-20YrBefore	0.0753	0.2364	64.50	27.59	13.36	0.0311	0.1771	0.4800	1.1290	3.0149
Water_Ave_0-10YrBefore	0.0048	0.0151	36.61	7.25	0.52	0.0018	0.0119	0.0306	0.0645	0.1793
Area_Ave_0-10YrBefore	0.0004	0.0020	36.61	0.17	0.00	0.0001	0.0008	0.0018	0.0042	0.0400
Maximum_0-10YrBefore	0.2353	0.4058	36.61	57.51	44.39	0.2791	0.6996	1.1871	1.7708	3.1358
Max_Ave_0-10YrBefore	0.1396	0.2527	36.61	55.44	35.58	0.1595	0.4391	0.6408	1.1434	2.0764
Water_Ave_0-5YrBefore	0.0063	0.0182	39.27	8.30	0.69	0.0025	0.0139	0.0453	0.0886	0.1584
Area_Ave_0-5YrBefore	0.0005	0.0027	39.27	0.17	0.00	0.0001	0.0011	0.0023	0.0061	0.0547
Maximum_0-5YrBefore	0.3060	0.4885	39.45	57.96	48.44	0.3906	0.9333	1.4528	1.9410	4.1951
Max_Ave_0-5YrBefore	0.1734	0.2862	39.45	56.75	40.83	0.2188	0.5166	0.8653	1.3130	1.6967
Water_Ave_0-5YrAfter	0.0076	0.0196	34.09	8.57	0.52	0.0056	0.0153	0.0450	0.0885	0.2378
Area_Ave_0-5YrAfter	0.0006	0.0031	34.09	0.35	0.00	0.0003	0.0014	0.0033	0.0064	0.0637
Maximum_0-5YrAfter	0.4171	0.5586	34.09	64.69	60.14	0.5664	1.1153	1.6901	2.1160	4.8308
Max_Ave_0-5YrAfter	0.2279	0.3020	34.09	64.51	55.42	0.3102	0.5636	0.9730	1.3214	1.8472
Water_Ave_Lifetime	0.0045	0.0130	27.24	6.90	0.34	0.0021	0.0112	0.0270	0.0637	0.1513
Area_Ave_Lifetime	0.0003	0.0014	27.24	0.17	0.00	0.0001	0.0007	0.0015	0.0045	0.0243
Maximum_Lifetime	0.2171	0.3432	27.41	67.07	48.79	0.2660	0.5902	0.8873	1.7660	2.3789
Max_Ave_Lifetime	0.1292	0.2180	27.41	64.48	34.14	0.1328	0.3587	0.5351	1.1164	1.8679

**Table 4:** Spearman correlation coefficients for individual-level cyanoHAB exposure measures for four concentration metrics, six exposure windows, and the 5 km buffer. Color coded from 0 to 1.0. (N = 584).

	Water_Ave_0-20YrBefore	Area_Ave_0-20YrBefore	Max_0-20YrBefore	Max_Ave_0-20YrBefore	Water_Ave_10-20YrBefore	Area_Ave_10-20YrBefore	Max_10-20YrBefore	Max_Ave_10-20YrBefore	Water_Ave_0-10YrBefore	Area_Ave_0-10YrBefore	Max_0-10YrBefore	Max_Ave_0-10YrBefore	Water_Ave_0-5YrBefore	Area_Ave_0-5YrBefore	Max_0-5YrBefore	Max_Ave_0-5YrBefore	Water_Ave_0-5YrAfter	Area_Ave_0-5YrAfter	Max_0-5YrAfter	Max_Ave_0-5YrAfter	Water_Ave_Lifetime	Area_Ave_Lifetime	Max_Lifetime	Max_Ave_Lifetime
**Water_Ave_0-20YrBefore**	1.00																							
**Area_Ave_0-20YrBefore**	0.95	1.00																			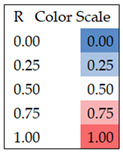			
**Max_0-20YrBefore**	0.95	0.96	1.00																					
**Max_Ave_0-20YrBefore**	0.96	0.92	0.98	1.00																				
**Water_Ave_10-20YrBefore**	0.69	0.71	0.72	0.70	1.00																			
**Area_Ave_10-20YrBefore**	0.66	0.73	0.72	0.68	0.98	1.00																		
**Max_10-20YrBefore**	0.67	0.73	0.75	0.71	0.96	0.98	1.00																	
**Max_Ave_10-20YrBefore**	0.68	0.72	0.75	0.72	0.96	0.96	0.99	1.00																
**Water_Ave_0-10YrBefore**	0.93	0.87	0.90	0.90	0.52	0.49	0.51	0.52	1.00															
**Area_Ave_0-10YrBefore**	0.89	0.92	0.91	0.86	0.53	0.54	0.57	0.56	0.95	1.00														
**Max_0-10YrBefore**	0.90	0.89	0.95	0.92	0.55	0.55	0.58	0.59	0.96	0.96	1.00													
**Max_Ave_0-10YrBefore**	0.90	0.83	0.91	0.94	0.52	0.50	0.53	0.54	0.96	0.90	0.97	1.00												
**Water_Ave_0-5YrBefore**	0.90	0.84	0.86	0.86	0.49	0.47	0.49	0.50	0.96	0.91	0.92	0.91	1.00											
**Area_Ave_0-5YrBefore**	0.85	0.89	0.88	0.83	0.50	0.52	0.54	0.54	0.91	0.97	0.93	0.86	0.94	1.00										
**Max_0-5YrBefore**	0.87	0.86	0.91	0.88	0.51	0.50	0.53	0.54	0.93	0.92	0.97	0.93	0.95	0.95	1.00									
**Max_Ave_0-5YrBefore**	0.86	0.78	0.86	0.89	0.47	0.44	0.46	0.48	0.92	0.84	0.92	0.96	0.95	0.86	0.95	1.00								
**Water_Ave_0-5YrAfter**	0.78	0.75	0.75	0.75	0.44	0.42	0.43	0.45	0.83	0.81	0.80	0.78	0.86	0.83	0.82	0.80	1.00							
**Area_Ave_0-5YrAfter**	0.75	0.80	0.77	0.72	0.46	0.47	0.49	0.49	0.79	0.86	0.80	0.74	0.81	0.88	0.81	0.73	0.93	1.00						
**Max_0-5YrAfter**	0.77	0.77	0.78	0.76	0.44	0.43	0.46	0.47	0.82	0.83	0.82	0.79	0.84	0.85	0.84	0.81	0.95	0.94	1.00					
**Max_Ave_0-5YrAfter**	0.72	0.64	0.69	0.72	0.36	0.33	0.36	0.37	0.77	0.70	0.74	0.78	0.79	0.71	0.76	0.81	0.90	0.78	0.90	1.00				
**Water_Ave_Lifetime**	0.96	0.91	0.91	0.91	0.63	0.61	0.62	0.63	0.91	0.86	0.86	0.87	0.88	0.84	0.84	0.84	0.88	0.83	0.85	0.80	1.00			
**Area_Ave_Lifetime**	0.91	0.97	0.92	0.88	0.66	0.68	0.68	0.68	0.85	0.91	0.86	0.80	0.82	0.89	0.84	0.76	0.82	0.88	0.83	0.69	0.94	1.00		
**Max_Lifetime**	0.93	0.94	0.97	0.94	0.66	0.66	0.69	0.69	0.90	0.91	0.93	0.90	0.87	0.89	0.91	0.86	0.85	0.86	0.88	0.79	0.95	0.95	1.00	
**Max_Ave_Lifetime**	0.93	0.87	0.93	0.96	0.63	0.61	0.64	0.65	0.89	0.84	0.89	0.92	0.86	0.81	0.87	0.89	0.84	0.78	0.85	0.85	0.95	0.88	0.96	1.00

**Table 5:** Covariate-adjusted Cox proportional hazards model results for ALS survival time since diagnosis in years for four exposure metrics, five buffer radii, and five exposure windows. Exposure windows show years before onset except “0–5”, which designates 0 to 5 years after disease onset. N = 307. Two cases with missing covariate data were excluded. Buffer radius in km. *p*- and Q-values ≤ 0.05 are shown in bold.

Exposure Window (Years)	Buf-fer Radius (km)	Water-Averaged Concentration			Area-Averaged Concentration			Maximum Concentration		Averaged Maximum Concentration			
		HR (95th CI)	*p*-Val-ue	Q-Val-ue (BH)	HR (95th CI)	*p*-Val-ue	Q-value (BH)	HR (95th CI)	*p*-Val-ue	Q-Val-ue (BH)	HR (95th CI)	*p*-Val-ue	Q-Value (BH)
0–5	0.5	1.05 (0.92, 1.19)	0.46	0.46	1.04 (0.92, 1.18)	0.50	0.50	1.05 (0.93, 1.19)	0.43	0.43	1.05 (0.93, 1.19)	0.42	0.47
years	1.0	1.08 (0.96, 1.21)	0.22	0.36	1.07 (0.95, 1.21)	0.26	0.43	1.09 (0.96, 1.23)	0.18	0.30	1.08 (0.96, 1.22)	0.20	0.34
before	2.5	1.12 (1.00, 1.27)	**0.06**	0.14	1.13 (1.00, 1.28)	**0.04**	0.10	1.15 (1.01, 1.30)	**0.03**	0.07	1.13 (1.00, 1.28)	**0.05**	0.12
onset	5.0	1.16 (1.02, 1.31)	**0.02**	0.12	1.16 (1.02, 1.31)	**0.02**	0.10	1.16 (1.02, 1.32)	**0.02**	0.07	1.15 (1.01, 1.30)	**0.04**	0.12
	10.0	1.07 (0.94, 1.22)	0.29	0.36	1.06 (0.93, 1.21)	0.35	0.44	1.06 (0.93, 1.20)	0.40	0.43	1.05 (0.92, 1.19)	0.47	0.47
0–10	0.5	1.06 (0.95, 1.18)	0.32	0.43	1.05 (0.94, 1.17)	0.41	0.41	1.06 (0.95, 1.18)	0.34	0.38	1.06 (0.95, 1.18)	0.32	0.37
years	1.0	1.05 (0.93, 1.19)	0.43	0.43	1.05 (0.93, 1.19)	0.41	0.41	1.06 (0.93, 1.19)	0.38	0.38	1.06 (0.94, 1.20)	0.37	0.37
before	2.5	1.16 (1.03, 1.30)	**0.01**	0.03	1.13 (1.01, 1.27)	**0.04**	0.09	1.17 (1.04, 1.32)	**0.01**	0.02	1.18 (1.04, 1.32)	**0.01**	0.02
onset	5.0	1.21 (1.07, 1.36)	**0.00**	0.01	1.19 (1.06, 1.34)	**0.00**	0.02	1.22 (1.08, 1.38)	**0.00**	0.01	1.23 (1.09, 1.38)	**0.01**	0.00
	10.0	1.05 (0.93, 1.19)	0.42	0.43	1.06 (0.94, 1.20)	0.31	0.41	1.10 (0.97, 1.24)	0.14	0.24	1.09 (0.97, 1.23)	0.16	0.27
10–20	0.5	1.05 (0.93, 1.20)	0.41	0.41	1.05 (0.93, 1.19)	0.46	0.53	1.06 (0.93, 1.20)	0.39	0.45	1.06 (0.93, 1.20)	0.38	0.48
years	1.0	1.09 (0.97, 1.23)	0.14	0.24	1.09 (0.96, 1.23)	0.17	0.29	1.09 (0.97, 1.23)	0.15	0.25	1.09 (0.96, 1.23)	0.17	0.28
before	2.5	1.17 (1.03, 1.32)	**0.01**	0.04	1.17 (1.04, 1.32)	**0.01**	0.04	1.18 (1.04, 1.34)	**0.01**	0.03	1.17 (1.04, 1.33)	**0.01**	0.05
onset	5.0	1.16 (1.03, 1.32)	**0.02**	0.04	1.16 (1.03, 1.32)	**0.02**	0.04	1.18 (1.03, 1.34)	**0.01**	0.03	1.17 (1.03, 1.33)	**0.02**	0.05
	10.0	1.05 (0.93, 1.19)	0.41	0.41	1.04 (0.92, 1.18)	0.53	0.53	1.05 (0.93, 1.19)	0.45	0.45	1.04 (0.92, 1.18)	0.50	0.50
0–20	0.5	1.05 (0.92, 1.19)	0.46	0.46	1.04 (0.92, 1.18)	0.50	0.50	1.05 (0.93, 1.19)	0.43	0.43	1.05 (0.93, 1.19)	0.42	0.47
years	1.0	1.08 (0.96, 1.21)	0.22	0.36	1.07 (0.95, 1.21)	0.26	0.43	1.09 (0.96, 1.23)	0.18	0.30	1.08 (0.96, 1.22)	0.20	0.34
before	2.5	1.12 (1.00, 1.27)	**0.06**	0.14	1.13 (1.00, 1.28)	**0.04**	0.10	1.15 (1.01, 1.30)	**0.03**	0.07	1.13 (1.00, 1.28)	**0.05**	0.12
onset	5.0	1.16 (1.02, 1.31)	**0.02**	0.12	1.16 (1.02, 1.31)	**0.02**	0.10	1.16 (1.02, 1.32)	**0.02**	0.07	1.15 (1.01, 1.30)	**0.04**	0.12
	10.0	1.07 (0.94, 1.22)	0.29	0.36	1.06 (0.93, 1.21)	0.35	0.44	1.06 (0.93, 1.20)	0.40	0.43	1.05 (0.92, 1.19)	0.47	0.47
0–5	0.5	0.94 (0.82, 1.08)	0.38	0.72	0.94 (0.82, 1.07)	0.37	0.61	0.94 (0.82, 1.08)	0.37	0.60	0.94 (0.82, 1.07)	0.36	0.68
years	1.0	0.94 (0.83, 1.07)	0.37	0.72	0.94 (0.82, 1.07)	0.34	0.61	0.94 (0.83, 1.07)	0.37	0.60	0.93 (0.82, 1.07)	0.31	0.68
after	2.5	1.01 (0.90, 1.15)	0.83	0.83	1.03 (0.91, 1.16)	0.69	0.69	1.05 (0.93, 1.19)	0.46	0.60	1.03 (0.91, 1.17)	0.60	0.68
onset	5.0	1.05 (0.93, 1.20)	0.43	0.72	1.07 (0.94, 1.21)	0.33	0.61	1.05 (0.92, 1.19)	0.48	0.60	1.03 (0.91, 1.17)	0.64	0.68
	10.0	1.03 (0.91, 1.17)	0.66	0.82	1.04 (0.91, 1.18)	0.60	0.69	1.03 (0.91, 1.18)	0.61	0.61	1.03 (0.90, 1.17)	0.68	0.68

**Table 6:** Covariate-adjusted Cox proportional hazard model results for ALS survival time (years) since diagnosis, with interactions for private well or city water supply. Shown are the results for a 5 km buffer with four concentration metrics and four exposure windows before onset. % Zero indicates the percentage of individuals without any cyanoHAB exposure. N = 236. Abbreviations: BH, Benjamini–Hochberg correction; CI, confidence interval; HR, hazard ratio. *p*- and Q-values ≤ 0.05 are shown in bold.

Concentra-tion Metric	Exposure Window (Year)	Private Well				City Water Supply			
		HR (95th CI)	*p*-Value	Q-Value (BH)	% Zero	HR (95th CI)	*p*-Value	Q-Value (BH)	% Zero
Maximum	0–5	1.31 (1.03, 1.66)	**0.026**	0.128	35.1%	1.14 (0.94, 1.37)	0.184	0.306	50.0%
	0–10	1.34 (1.05, 1.70)	**0.017**	0.083	33.0%	1.15 (0.96, 1.39)	0.139	0.231	49.3%
	10–20	1.23 (1.00, 1.53)	0.053	0.132	60.8%	1.20 (1.01, 1.43)	**0.040**	0.200	71.9%
	0–20	1.44 (1.13, 1.84)	**0.003**	**0.016**	27.8%	1.16 (0.97, 1.39)	0.113	0.253	46.8%
Area-	0–5	1.26 (0.99, 1.59)	0.056	0.278	35.1%	1.17 (0.97, 1.40)	0.098	0.310	50.0%
Average	0–10	1.27 (1.01, 1.60)	**0.042**	0.141	33.0%	1.18 (0.99, 1.40)	0.067	0.209	49.3%
	10–20	1.22 (0.99, 1.51)	0.065	0.187	60.8%	1.17 (0.99, 1.38)	0.071	0.354	72.7%
	0–20	1.39 (1.10, 1.75)	**0.006**	**0.029**	27.8%	1.18 (0.99, 1.41)	0.059	0.266	46.8%
Water-	0–5	1.32 (1.04, 1.67)	**0.022**	0.108	35.1%	1.14 (0.95, 1.37)	0.154	0.319	50.0%
Average	0–10	1.35 (1.06, 1.71)	**0.013**	0.067	33.0%	1.15 (0.96, 1.38)	0.124	0.207	49.3%
	10–20	1.24 (1.00, 1.54)	0.051	0.127	60.8%	1.17 (0.99, 1.39)	0.067	0.335	72.7%
	0–20	1.47 (1.15, 1.88)	**0.002**	**0.011**	27.8%	1.15 (0.96, 1.38)	0.125	0.265	46.8%
Maximum	0–5	1.33 (1.05, 1.68)	**0.018**	0.091	35.1%	1.12 (0.92, 1.35)	0.253	0.365	50.0%
Average	0–10	1.35 (1.06, 1.71)	**0.014**	0.069	33.0%	1.14 (0.94, 1.37)	0.180	0.300	49.3%
	10–20	1.24 (1.00, 1.53)	0.054	0.148	60.8%	1.21 (1.01, 1.44)	**0.037**	0.187	71.9%
	0–20	1.45 (1.14, 1.86)	**0.003**	**0.013**	27.8%	1.14 (0.95, 1.37)	0.156	0.256	46.8%

**Table 7:** Covariate-adjusted Cox proportional hazards model results for ALS survival time (years) since diagnosis with interactions for fishing/swimming. Shown are the results for a 5 km buffer with four concentration metrics and four exposure windows before onset. % Zero indicates the percentage of individuals without any cyanoHAB exposure. *p*- and Q-values ≤ 0.05 are shown in bold. N = 243. Abbreviations: BH, Benjamini–Hochberg correction; CI, confidence interval; HR, hazard ratio.

Concentration Metric	Exposure Window (Year)	Fishing/Swimming				No Fishing/Swimming			
		HR (95th CI)	*p*-Value	Q-Value (BH)	% Zero	HR (95th CI)	*p*-Value	Q-Value (BH)	% Zero
Maximum	0–5	1.31 (1.09, 1.57)	**0.004**	**0.021**	48.1%	1.08 (0.85, 1.37)	0.540	0.735	35.6%
	0–10	1.34 (1.11, 1.60)	**0.002**	**0.006**	45.5%	1.07 (0.85, 1.36)	0.561	0.702	35.6%
	10–20	1.28 (1.07, 1.53)	**0.006**	**0.029**	67.5%	1.15 (0.93, 1.42)	0.210	0.417	65.9%
	0–20	1.38 (1.15, 1.65)	**<0.001**	**0.002**	42.9%	1.08 (0.86, 1.37)	0.500	0.824	31.8%
Area-	0–5	1.29 (1.08, 1.53)	**0.005**	**0.024**	48.1%	1.09 (0.86, 1.38)	0.485	0.608	35.6%
average	0–10	1.30 (1.10, 1.55)	**0.003**	**0.011**	45.5%	1.10 (0.87, 1.39)	0.431	0.657	35.6%
	10–20	1.24 (1.05, 1.47)	**0.012**	0.060	68.2%	1.12 (0.91, 1.39)	0.286	0.477	65.9%
	0–20	1.36 (1.14, 1.61)	**<0.001**	**0.003**	42.9%	1.10 (0.87, 1.39)	0.413	0.689	31.8%
Water-	0–5	1.31 (1.09, 1.57)	**0.004**	**0.020**	48.1%	1.10 (0.87, 1.38)	0.430	0.574	35.6%
average	0–10	1.33 (1.11, 1.60)	**0.002**	**0.010**	45.5%	1.10 (0.87, 1.37)	0.426	0.622	35.6%
	10–20	1.26 (1.06, 1.51)	**0.009**	**0.043**	68.2%	1.13 (0.92, 1.39)	0.255	0.504	65.9%
	0–20	1.38 (1.15, 1.66)	**<0.001**	**0.002**	42.9%	1.09 (0.86, 1.37)	0.479	0.761	31.8%
Annual	0–5	1.30 (1.08, 1.57)	**0.005**	**0.026**	48.1%	1.07 (0.85, 1.35)	0.562	0.688	35.6%
Maximum	0–10	1.34 (1.12, 1.61)	**0.002**	**0.007**	45.5%	1.06 (0.84, 1.34)	0.618	0.670	35.6%
	10–20	1.30 (1.09, 1.56)	**0.004**	**0.020**	67.5%	1.14 (0.92, 1.41)	0.224	0.451	65.9%
	0–20	1.38 (1.15, 1.66)	**<0.001**	**0.003**	42.9%	1.07 (0.85, 1.35)	0.562	0.776	31.8%

## Data Availability

Identifiable data cannot be shared. Sharing of non-identifiable data will be considered at the reasonable request of a qualified investigator.

## Supplementary Materials

The following supporting information can be downloaded at: https://www.mdpi.com/article/10.3390/ijerph22050763/s1. The SI provides additional information regarding distribution of cyanoHAB exposure estimates at the residence level for water-averaged concentrations and for various sized buffers (Figures S1 and S2); residence-level log probability plots for select years and the water-averaged concentration metric with 5 km buffer (Figure S3), correlation coefficients for HAB concentrations at the residential level for 2005 (Table S1), descriptive statistics for cyanoHAB concentrations (Table S2), demographics of ALS cases (Table S3), unadjusted Cox proportional hazards model results (Table S4), survival curves for four concentration metrics and low and high exposure groups (Figure S4), Cox, Kaplan–Meier, and Royston–Parmar survival curves (Figure S5), survival curves for the 0–20-year exposure window using exposure quartiles (Figure S6), Cox proportional hazards model results for ALS survival time (years) since diagnosis stratified by private well or city water supply (Table S5), survival curves comparing upper and lower exposure groups for interactions with water source (Figure S7), Cox proportional hazards model results for ALS survival time (years) since diagnosis stratified by fishing/swimming (Table S6), and survival curves comparing upper and lower exposure groups for interactions with fishing and swimming (Figure S8).

## References

[1] Anderson D.M., Backer L., Bouma-Gregson K., Bowers H.A., Bricelj V., D’Anglada L.V., Deeds J., Dortch Q., Doucette G.J., Graham J. (2024). Harmful Algal Research & Response: A National Environmental Science Strategy (HARRNESS), 2024–2034.

[2] Chatterjee S., More M. (2023). Cyanobacterial Harmful Algal Bloom Toxin Microcystin and Increased Vibrio Occurrence as Climate-Change-Induced Biological Co-Stressors: Exposure and Disease Outcomes via Their Interaction with Gut–Liver–Brain Axis. Toxins.

[3] Kazmi S.S.U.H., Kazmi S.S.U.H., Yapa N., Yapa N., Karunarathna S.C., Karunarathna S.C., Suwannarach N., Suwannarach N. (2022). Perceived Intensification in Harmful Algal Blooms Is a Wave of Cumulative Threat to the Aquatic Ecosystems. Biology.

[4] Sayers M.J., Grimm A.G., Shuchman R.A., Bosse K.R., Fahnenstiel G.L., Ruberg S.A., Leshkevich G.A. (2019). Satellite Monitoring of Harmful Algal Blooms in the Western Basin of Lake Erie: A 20-Year Time-Series. J. Great Lakes Res..

[5] Metcalf J.S., Tischbein M., Cox P.A., Stommel E.W. (2021). Cyanotoxins and the Nervous System. Toxins.

[6] Nugumanova G., Ponomarev E.D., Askarova S., Fasler-Kan E., Barteneva N.S. (2023). Freshwater Cyanobacterial Toxins, Cyanopeptides and Neurodegenerative Diseases. Toxins.

[7] Pablo J., Banack S.A., Cox P.A., Johnson T.E., Papapetropoulos S., Bradley W.G., Buck A., Mash D.C. (2009). Cyanobacterial Neurotoxin BMAA in ALS and Alzheimer’s Disease. Acta Neurol. Scand..

[8] Clark J.M., Schaeffer B.A., Darling J.A., Urquhart E.A., Johnston J.M., Ignatius A.R., Myer M.H., Loftin K.A., Werdell P.J., Stumpf R.P. (2017). Satellite Monitoring of Cyanobacterial Harmful Algal Bloom Frequency in Recreational Waters and Drinking Water Sources. Ecol. Indic..

[9] Plaas H.E., Paerl H.W. (2021). Toxic Cyanobacteria: A Growing Threat to Water and Air Quality. Environ. Sci. Technol..

[10] Backer L.C., Manassaram-Baptiste D., LePrell R., Bolton B. (2015). Cyanobacteria and Algae Blooms: Review of Health and Environmental Data from the Harmful Algal Bloom-Related Illness Surveillance System (HABISS) 2007–2011. Toxins.

[11] Lad A., Breidenbach J.D., Su R.C., Murray J., Kuang R., Mascarenhas A., Najjar J., Patel S., Hegde P., Youssef M. (2022). As We Drink and Breathe: Adverse Health Effects of Microcystins and Other Harmful Algal Bloom Toxins in the Liver, Gut, Lungs and Beyond. Life.

[12] Gorham T., Dowling Root E., Jia Y., Shum C.K., Lee J. (2020). Relationship between Cyanobacterial Bloom Impacted Drinking Water Sources and Hepatocellular Carcinoma Incidence Rates. Harmful Algae.

[13] Zhang F., Lee J., Liang S., Shum C. (2015). Cyanobacteria Blooms and Non-Alcoholic Liver Disease: Evidence from a County Level Ecological Study in the United States. Environ. Health.

[14] Lee S., Kim J., Choi B., Kim G., Lee J. (2019). Harmful Algal Blooms and Liver Diseases: Focusing on the Areas near the Four Major Rivers in South Korea. J. Environ. Sci. Health Part C.

[15] Li Y., Chen J., Zhao Q., Pu C., Qiu Z., Zhang R., Shu W. (2011). A Cross-Sectional Investigation of Chronic Exposure to Microcystin in Relationship to Childhood Liver Damage in the Three Gorges Reservoir Region, China. Environ. Health Perspect..

[16] Svirčev Z., Drobac D., Tokodi N., Lužanin Z., Munjas A.M., Nikolin B., Vuleta D., Meriluoto J. (2014). Epidemiology of Cancers in Serbia and Possible Connection with Cyanobacterial Blooms. J. Environ. Sci. Health Part C.

[17] Torbick N., Ziniti B., Stommel E., Linder E., Andrew A., Caller T., Haney J., Bradley W., Henegan P.L., Shi X. (2018). Assessing Cyanobacterial Harmful Algal Blooms as Risk Factors for Amyotrophic Lateral Sclerosis. Neurotox. Res..

[18] Whitton B.A. (2012). Ecology of Cyanobacteria II: Their Diversity in Space and Time.

[19] Stommel E.W., Field N.C., Caller T.A. (2013). Aerosolization of Cyanobacteria as a Risk Factor for Amyotrophic Lateral Sclerosis. Med. Hypotheses.

[20] Sakowski S.A., Koubek E.J., Chen K.S., Goutman S.A., Feldman E.L. (2024). Role of the Exposome in Neurodegenerative Disease: Recent Insights and Future Directions. Ann. Neurol..

[21] Davis D.A., Cox P.A., Banack S.A., Lecusay P.D., Garamszegi S.P., Hagan M.J., Powell J.T., Metcalf J.S., Palmour R.M., Beierschmitt A. (2020). L-Serine Reduces Spinal Cord Pathology in a Vervet Model of Preclinical ALS/MND. J. Neuropathol. Exp. Neurol..

[22] Rao S.D., Banack S.A., Cox P.A., Weiss J.H. (2006). BMAA Selectively Injures Motor Neurons via AMPA/Kainate Receptor Activation. Exp. Neurol..

[23] Lobner D., Piana P.M.T., Salous A.K., Peoples R.W. (2007). Beta-N-Methylamino-L-Alanine Enhances Neurotoxicity through Multiple Mechanisms. Neurobiol. Dis..

[24] Goutman S.A., Hardiman O., Al-Chalabi A., Chió A., Savelieff M.G., Kiernan M.C., Feldman E.L. (2022). Emerging Insights into the Complex Genetics and Pathophysiology of Amyotrophic Lateral Sclerosis. Lancet Neurol..

[25] Goutman S.A., Savelieff M.G., Jang D.-G., Hur J., Feldman E.L. (2023). The Amyotrophic Lateral Sclerosis Exposome: Recent Advances and Future Directions. Nat. Rev. Neurol..

[26] Dou J., Bakulski K., Guo K., Hur J., Zhao L., Saez-Atienzar S., Stark A., Chia R., García-Redondo A., Rojas-Garcia R. (2023). Cumulative Genetic Score and C9orf72 Repeat Status Independently Contribute to Amyotrophic Lateral Sclerosis Risk in 2 Case-Control Studies. Neurol. Genet..

[27] Goutman S.A., Boss J., Godwin C., Mukherjee B., Feldman E.L., Batterman S.A. (2023). Occupational History Associates with ALS Survival and Onset Segment. Amyotroph. Lateral Scler. Front. Degener..

[28] Goutman S.A., Boss J., Jang D.-G., Mukherjee B., Richardson R.J., Batterman S., Feldman E.L. (2023). Environmental Risk Scores of Persistent Organic Pollutants Associate with Higher ALS Risk and Shorter Survival in a New Michigan Case/Control Cohort. J. Neurol. Neurosurg. Psychiatry.

[29] Goutman S.A., Boss J., Jang D.G., Piecuch C., Farid H., Batra M., Mukherjee B., Feldman E.L., Batterman S.A. (2024). Avocational Exposure Associations with ALS Risk, Survival, and Phenotype: A Michigan-Based Case-Control Study. J. Neurol. Sci..

[30] Goutman S.A., Boss J., Jang D.G., Piecuch C., Farid H., Batra M., Mukherjee B., Feldman E.L., Batterman S.A. (2024). Residential Exposure Associations with ALS Risk, Survival, and Phenotype: A Michigan-Based Case-Control Study. Amyotroph. Lateral Scler. Front. Degener..

[31] Zhao Y., Li X., Wang K., Iyer G., Sakowski S.A., Zhao L., Teener S., Bakulski K.M., Dou J.F., Traynor B.J. (2024). Epigenetic Age Acceleration Is Associated with Occupational Exposures, Sex, and Survival in Amyotrophic Lateral Sclerosis. EBioMedicine.

[32] Shefner J.M., Al-Chalabi A., Baker M.R., Cui L.-Y., De Carvalho M., Eisen A., Grosskreutz J., Hardiman O., Henderson R., Matamala J.M. (2020). A Proposal for New Diagnostic Criteria for ALS. Clin. Neurophysiol..

[33] Goutman S.A., Boss J., Godwin C., Mukherjee B., Feldman E.L., Batterman S.A. (2022). Associations of Self-Reported Occupational Exposures and Settings to ALS: A Case–Control Study. Int. Arch. Occup. Environ. Health.

[34] Chaffin J.D., Bratton J.F., Verhamme E.M., Bair H.B., Beecher A.A., Binding C.E., Birbeck J.A., Bridgeman T.B., Chang X., Crossman J. (2021). The Lake Erie HABs Grab: A Binational Collaboration to Characterize the Western Basin Cyanobacterial Harmful Algal Blooms at an Unprecedented High-Resolution Spatial Scale. Harmful Algae.

[35] French B.W., Kaul R., George J., Haller S., Kennedy D.J., Mukundan D. (2023). A Case Series of Potential Pediatric Cyanotoxin Exposures Associated with Harmful Algal Blooms in Northwest Ohio. Infect. Dis. Rep..

[36] Backer L.C., Backer L.C. (2012). Freshwater Algal Blooms & Public Health. Lake Line.

[37] Zorbas V., Jang M., Emam B., Choi J. (2023). Modeling of the Atmospheric Process of Cyanobacterial Toxins in Algal Aerosol. ACS Earth Space Chem..

[38] Lunetta R.S., Schaeffer B.A., Stumpf R.P., Keith D., Jacobs S.A., Murphy M.S. (2015). Evaluation of Cyanobacteria Cell Count Detection Derived from MERIS Imagery across the Eastern USA. Remote Sens. Environ..

[39] Jang M., Berthold D.E., Yu Z., Silva-Sanchez C., Laughinghouse Iv H.D., Denslow N.D., Han S. (2020). Atmospheric Progression of Microcystin-LR from Cyanobacterial Aerosols. Environ. Sci. Technol. Lett..

[40] Schaeffer B., Loftin K., Stumpf R., Werdell P. (2015). Agencies Collaborate, Develop a Cyanobacteria Assessment Network. Eos.

[41] Handler A.M., Compton J.E., Hill R.A., Leibowitz S.G., Schaeffer B.A. (2023). Identifying Lakes at Risk of Toxic Cyanobacterial Blooms Using Satellite Imagery and Field Surveys across the United States. Sci. Total Environ..

[42] Goutman S.A., Boss J., Patterson A., Mukherjee B., Batterman S., Feldman E.L. (2019). High Plasma Concentrations of Organic Pollutants Negatively Impact Survival in Amyotrophic Lateral Sclerosis. J. Neurol. Neurosurg. Psychiatry.

[43] Murdock B.J., Goutman S.A., Boss J., Kim S., Feldman E.L. (2021). Amyotrophic Lateral Sclerosis Survival Associates with Neutrophils in a Sex-Specific Manner. Neurol. Neuroimmunol. Neuroinflamm.

[44] Shi J.H., Olson N.E., Birbeck J.A., Pan J., Peraino N.J., Holen A.L., Ledsky I.R., Jacquemin S.J., Marr L.C., Schmale D.G. (2023). Aerosolized Cyanobacterial Harmful Algal Bloom Toxins: Microcystin Congeners Quantified in the Atmosphere. Environ. Sci. Technol..

[45] Stumpf R.P., Davis T.W., Wynne T.T., Graham J.L., Loftin K.A., Johengen T.H., Gossiaux D., Palladino D., Burtner A. (2016). Challenges for Mapping Cyanotoxin Patterns from Remote Sensing of Cyanobacteria. Harmful Algae.

[46] Liu Q., Rowe M.D., Anderson E.J., Stow C.A., Stumpf R.P., Johengen T.H. (2020). Probabilistic Forecast of Microcystin Toxin Using Satellite Remote Sensing, in Situ Observations and Numerical Modeling. Environ. Model. Softw..

[47] Ai Y., Lee S., Lee J. (2020). Drinking Water Treatment Residuals from Cyanobacteria Bloom-Affected Areas: Investigation of Potential Impact on Agricultural Land Application. Sci. Total Environ..

[48] Brender J.D., Maantay J.A., Chakraborty J. (2011). Residential Proximity to Environmental Hazards and Adverse Health Outcomes. Am. J. Public Health.

[49] Zandbergen P.A., Green J.W. (2007). Error and Bias in Determining Exposure Potential of Children at School Locations Using Proximity-Based GIS Techniques. Environ. Health Perspect..

[50] Zandbergen P.A., Chakraborty J. (2006). Improving Environmental Exposure Analysis Using Cumulative Distribution Functions and Individual Geocoding. Int. J. Health Geogr..

[51] Su J.G., Jerrett M., Beckerman B., Wilhelm M., Ghosh J.K., Ritz B. (2009). Predicting Traffic-Related Air Pollution in Los Angeles Using a Distance Decay Regression Selection Strategy. Environ. Res..

[52] Su F.-C., Goutman S.A., Chernyak S., Mukherjee B., Callaghan B.C., Batterman S., Feldman E.L. (2016). Association of Environmental Toxins with Amyotrophic Lateral Sclerosis. JAMA Neurol..

[53] Figueroa-Romero C., Mikhail K.A., Gennings C., Curtin P., Bello G.A., Botero T.M., Goutman S.A., Feldman E.L., Arora M., Austin C. (2020). Early Life Metal Dysregulation in Amyotrophic Lateral Sclerosis. Ann. Clin. Transl. Neurol..

[54] Jang D.G., Dou J., Koubek E.J., Teener S., Zhao L., Bakulski K.M., Mukherjee B., Batterman S.A., Feldman E.L., Goutman S.A. (2024). Metal Mixtures Associate with Higher Amyotrophic Lateral Sclerosis Risk and Mortality Independent of Genetic Risk and Correlate to Self-Reported Exposures: A Case-Control Study. medRxiv.

[55] Spencer P.S., Palmer V.S., Kisby G.E. (2020). Western Pacific ALS-PDC: Evidence Implicating Cycad Genotoxins. J. Neurol. Sci..

[56] Chernoff N., Hill D.J., Diggs D.L., Faison B.D., Francis B.M., Lang J.R., Larue M.M., Le T.-T., Loftin K.A., Lugo J.N. (2017). A Critical Review of the Postulated Role of the Non-Essential Amino Acid, β-N-Methylamino-L-Alanine, in Neurodegenerative Disease in Humans. J. Toxicol. Environ. Health B Crit. Rev..

[57] Cox P.A., Banack S.A., Murch S.J. (2003). Biomagnification of Cyanobacterial Neurotoxins and Neurodegenerative Disease among the Chamorro People of Guam. Proc. Natl. Acad. Sci. USA.

[58] Berntzon L., Ronnevi L.O., Bergman B., Eriksson J. (2015). Detection of BMAA in the Human Central Nervous System. Neuroscience.

[59] Banack S.A., Caller T., Henegan P., Haney J., Murby A., Metcalf J.S., Powell J., Cox P.A., Stommel E. (2015). Detection of Cyanotoxins, β-N-Methylamino-L-Alanine and Microcystins, from a Lake Surrounded by Cases of Amyotrophic Lateral Sclerosis. Toxins.

[60] Metcalf J.S., Banack S.A., Lindsay J., Morrison L.F., Cox P.A., Codd G.A. (2008). Co-Occurrence of Beta-N-Methylamino-L-Alanine, a Neurotoxic Amino Acid with Other Cyanobacterial Toxins in British Waterbodies, 1990-2004. Environ. Microbiol..

[61] Caller T.A., Doolin J.W., Haney J.F., Murby A.J., West K.G., Farrar H.E., Ball A., Harris B.T., Stommel E.W. (2009). A Cluster of Amyotrophic Lateral Sclerosis in New Hampshire: A Possible Role for Toxic Cyanobacteria Blooms. Amyotroph. Lateral Scler..

[62] Torbick N., Hession S., Stommel E., Caller T. (2014). Mapping Amyotrophic Lateral Sclerosis Lake Risk Factors across Northern New England. Int. J. Health Geogr..

[63] Fiore M., Parisio R., Filippini T., Mantione V., Platania A., Odone A., Signorelli C., Pietrini V., Mandrioli J., Teggi S. (2020). Living near Waterbodies as a Proxy of Cyanobacteria Exposure and Risk of Amyotrophic Lateral Sclerosis: A Population Based Case-Control Study. Environ. Res..

[64] Mehta P., Raymond J., Nair T., Han M., Punjani R., Larson T., Berry J., Mohidul S., Horton D.K. (2024). Prevalence of ALS in All 50 States in the United States, Data from the National ALS Registry, 2011–2018. Amyotroph Lateral Scler Front. Degener.

