# Supplementary material for: Life Course Exposure to Cyanobacteria and Amyotrophic Lateral Sclerosis Survival
Source: Int J Environ Res Public Health. 2025 May 12;22(5):763. doi: 10.3390/ijerph22050763 (PMC12110991; doi:10.3390/ijerph22050763)
Supplement: Supplementary file 1 [file ijerph-22-00763-s001.zip › ijerph-3591108-supplementary.pdf]

Life course exposure to cyanobacteria and ALS survival

Stuart A. Batterman, Md Kamrul Islam, Dae Gyu Jang, Eva L. Feldman, Stephen A. Goutman

Supplemental Information

March 27, 2025

Contents

Figure S1 Log probability plot for cyanoHAB exposure at the residence level for water-averaged concentrations and 0.25, 0.5, 1, 2.5, 5, and 10 km buffers. 2016-2023 average. N=3380. .... S2

Figure S2. Residence-level log probability plots for selected years and water-averaged concentration metric using 5 km buffer. N=3380 residences. S2

Table S1. Spearman correlation coefficients for HAB concentrations for 3380 residential locations and year 2005. .... S3

Figure S3. Effect of buffer size on HAB exposure showing probability of HAB detections at the individual level (cases) for six exposure windows (N=322) S3

Table S2A. Descriptive statistics for cyanobacteria concentrations at the individual level for five buffer sizes, six exposure windows and three concentration metrics. S4

Table S2B. Descriptive statistics for cyanobacteria concentrations for five buffer sizes, six exposure windows and three concentration metrics. Otherwise as Table S2A S5

Table S2C. Descriptive statistics for cyanobacteria concentrations for five buffer sizes, six exposure windows and three concentration metrics. Otherwise as Table S2A S6

Table S3. Demographic characteristics of ALS study participants. Number, percentage or median (with interquartile range in parentheses). S7

Table S4. Unadjusted Cox proportional hazards model results for ALS survival time since diagnosis in years for 5 exposure windows, 5 buffer radii, and 4 exposure metrics. N=307. .... S8

Figure S4. Survival curves for four concentration metrics and low and high exposure groups. S9

Figure S5. Cox, Kaplan-Meier, and Royston Parmer survival curves. S9

Figure S6. Survival curves for the 0-20 year exposure window using exposure quartiles. S10

Table S5. Cox proportional hazards model results for ALS survival time (years) since diagnosis stratified by private well or city water supply. S11

Figure S7. Survival curves comparing upper and lower exposure groups for interactions with water source. S12

Table S6. Cox proportional hazards model results for ALS survival time (years) since diagnosis stratified by fishing/swimming. S13

Figure S8. Survival curves comparing upper and lower exposure groups for interactions with fishing and swimming S14

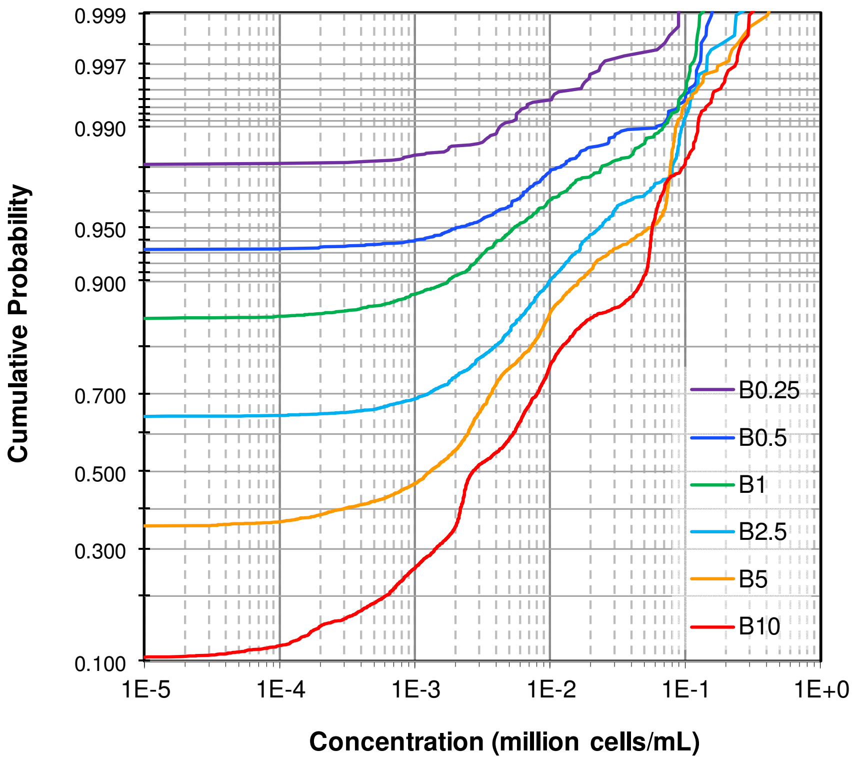

Figure S1 Log probability plot for cyanoHAB exposure at the residence level for water-averaged concentrations and 0.25, 0.5, 1, 2.5, 5, and 10 km buffers. 2016-2023 average. N=3380.

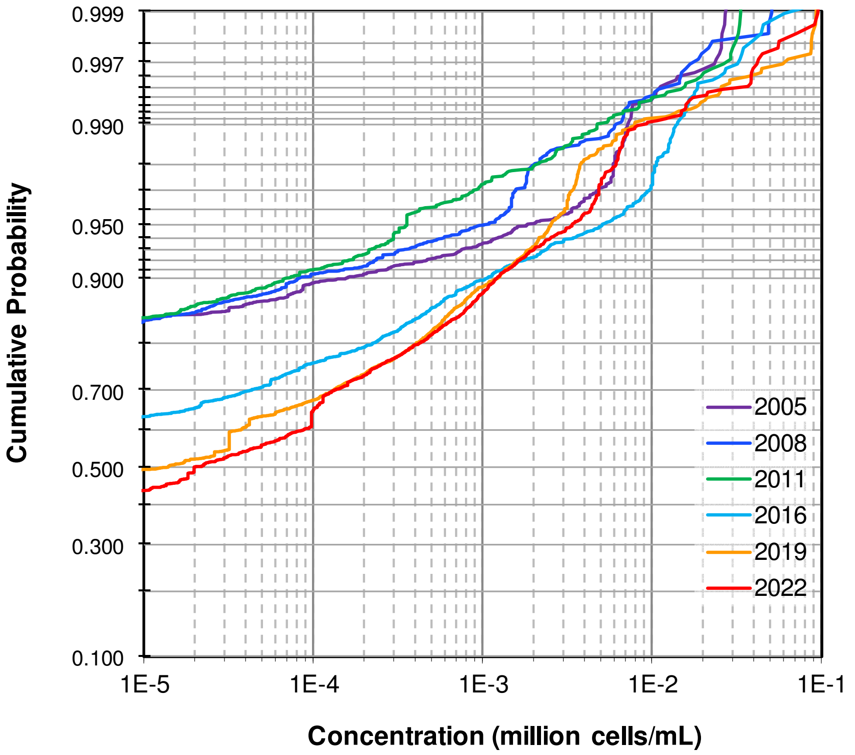

Figure S2. Residence-level log probability plots for selected years and water-averaged concentration metric using 5 km buffer. N=3380 residences.

Table S1. Spearman correlation coefficients for HAB concentrations for 3380 residential locations and year 2005.

	B250_A_Ave	B250_W_Ave	B250_Max	B250_Amax	B500_A_Ave	B500_W_Ave	B500_Max	B500_Amax	B01_A_Ave	B01_W_Ave	B01_Max	B01_Amax	B2.5_A_Ave	B2.5_W_Ave	B2.5_Max	B2.5_Amax	B05_A_Ave	B05_W_Ave	B05_Max	B05_Amax	B10_A_Ave	B10_W_Ave	B10_Max	B10_Amax
**B250_A_Ave**	1.00																							
**B250_W_Ave**	0.95	1.00																						
**B250_Max**	0.95	1.00	1.00																					
**B250_Amax**	0.94	0.99	1.00	1.00																				
**B500_A_Ave**	0.55	0.55	0.55	0.54	1.00																			
**B500_W_Ave**	0.49	0.51	0.51	0.50	0.98	1.00																		
**B500_Max**	0.51	0.52	0.52	0.52	0.96	0.97	1.00																	
**B500_Amax**	0.49	0.50	0.50	0.50	0.96	0.98	0.99	1.00																
**B01_A_Ave**	0.37	0.38	0.38	0.37	0.77	0.79	0.77	0.77	1.00															
**B01_W_Ave**	0.34	0.34	0.34	0.34	0.73	0.74	0.72	0.73	0.99	1.00														
**B01_Max**	0.36	0.37	0.37	0.37	0.72	0.73	0.73	0.73	0.98	0.98	1.00													
**B01_Amax**	0.35	0.35	0.35	0.35	0.70	0.71	0.72	0.72	0.97	0.98	0.99	1.00												
**B2.5_A_Ave**	0.24	0.24	0.24	0.24	0.50	0.50	0.49	0.50	0.68	0.68	0.68	0.67	1.00											
**B2.5_W_Ave**	0.21	0.21	0.21	0.21	0.43	0.44	0.43	0.44	0.59	0.60	0.59	0.59	0.96	1.00										
**B2.5_Max**	0.23	0.23	0.23	0.23	0.44	0.44	0.45	0.45	0.60	0.61	0.61	0.61	0.96	0.98	1.00									
**B2.5_Amax**	0.22	0.21	0.21	0.21	0.41	0.42	0.42	0.42	0.55	0.56	0.56	0.57	0.94	0.98	0.99	1.00								
**B05_A_Ave**	0.15	0.15	0.15	0.15	0.33	0.33	0.33	0.33	0.45	0.45	0.44	0.44	0.73	0.74	0.74	0.73	1.00							
**B05_W_Ave**	0.14	0.14	0.14	0.14	0.28	0.28	0.28	0.28	0.38	0.38	0.38	0.38	0.67	0.70	0.69	0.69	0.98	1.00						
**B05_Max**	0.14	0.14	0.14	0.14	0.28	0.28	0.28	0.28	0.38	0.38	0.39	0.39	0.64	0.66	0.68	0.67	0.97	0.98	1.00					
**B05_Amax**	0.12	0.12	0.12	0.12	0.24	0.24	0.24	0.24	0.33	0.33	0.34	0.34	0.61	0.64	0.64	0.65	0.95	0.97	0.98	1.00				
**B10_A_Ave**	0.10	0.10	0.10	0.10	0.22	0.22	0.21	0.22	0.29	0.29	0.29	0.28	0.47	0.48	0.48	0.47	0.68	0.67	0.67	0.65	1.00			
**B10_W_Ave**	0.09	0.08	0.08	0.08	0.18	0.18	0.17	0.18	0.23	0.23	0.23	0.23	0.39	0.42	0.41	0.41	0.60	0.62	0.60	0.60	0.96	1.00		
**B10_Max**	0.09	0.09	0.09	0.09	0.18	0.18	0.18	0.18	0.24	0.24	0.24	0.24	0.40	0.41	0.42	0.42	0.62	0.63	0.64	0.63	0.95	0.94	1.00	
**B10_Amax**	0.07	0.07	0.07	0.07	0.14	0.15	0.14	0.15	0.19	0.19	0.19	0.20	0.33	0.35	0.35	0.36	0.53	0.56	0.56	0.58	0.90	0.94	0.95	1.00

R	Color Scale
0.00	0.00
0.25	0.25
0.50	0.50
0.75	0.75
1.00	1.00

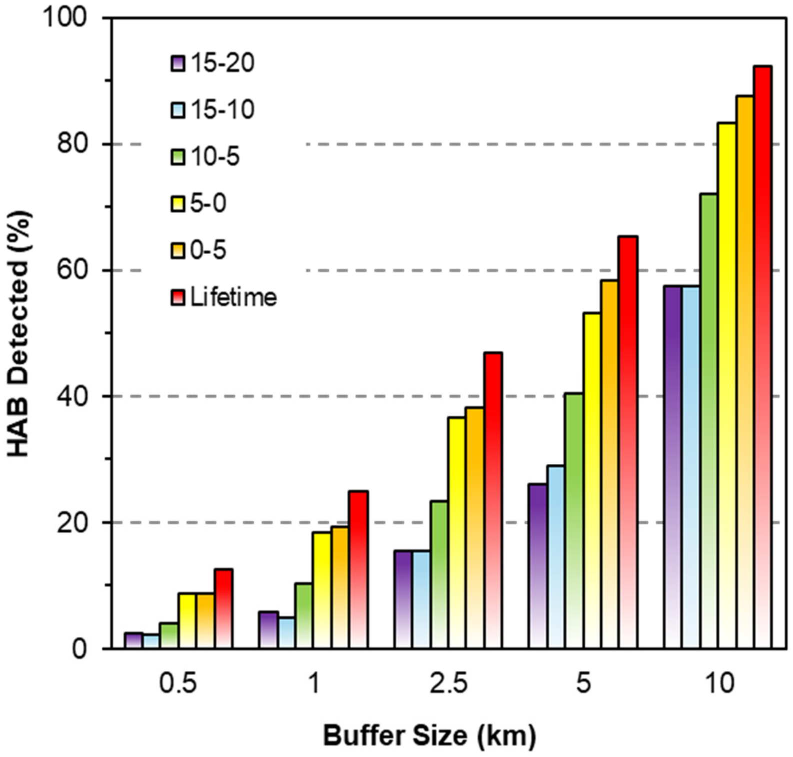

Figure S3. Effect of buffer size on HAB exposure showing probability of HAB detections at the individual level (cases) for six exposure windows (N=322).

Table S2A. Descriptive statistics for cyanobacteria concentrations at the individual level for five buffer sizes, six exposure windows and three concentration metrics.
N=584. Buffer sizes are: 250 m (B250), 500 m (B500), 1 km (B01), 5 km (B05), 10 km (B10). Yr, year. Before references date of onset (cases) or enrollment (controls).

Type	Variable	%zero	Mean	SD	Min	Q10	Q25	Q50	Q75	Q90	Q95	Max
Area Average	B250_a_ave_15-20yrBefore	94.3	0.000007	0.000121	0.000000	0.000000	0.000000	0.000000	0.000000	0.000000	0.000000	0.002690
	B250_a_ave_10-15yrBefore	96.1	0.000046	0.000819	0.000000	0.000000	0.000000	0.000000	0.000000	0.000000	0.000000	0.018856
	B250_a_ave_5-10yrBefore	96.4	0.000062	0.000954	0.000000	0.000000	0.000000	0.000000	0.000000	0.000000	0.000000	0.018083
	B250_a_ave_0-5yrBefore	96.2	0.000154	0.001657	0.000000	0.000000	0.000000	0.000000	0.000000	0.000000	0.000000	0.027472
	B250_a_ave_10-20yrBefore	96.4	0.000065	0.001348	0.000000	0.000000	0.000000	0.000000	0.000000	0.000000	0.000000	0.031948
	B250_a_ave_0-10yrBefore	96.2	0.000109	0.001050	0.000000	0.000000	0.000000	0.000000	0.000000	0.000000	0.000000	0.013836
	B250_a_ave_0-5yrAfter	95.5	0.000126	0.001110	0.000000	0.000000	0.000000	0.000000	0.000000	0.000000	0.000000	0.014311
	B500_a_ave_15-20yrBefore	93.2	0.000069	0.000945	0.000000	0.000000	0.000000	0.000000	0.000000	0.000000	0.000000	0.017621
	B500_a_ave_10-15yrBefore	94.7	0.000149	0.001648	0.000000	0.000000	0.000000	0.000000	0.000000	0.000000	0.000000	0.026647
	B500_a_ave_5-10yrBefore	93.8	0.000199	0.002174	0.000000	0.000000	0.000000	0.000000	0.000000	0.000000	0.000000	0.038591
	B500_a_ave_10-20yrBefore	95.0	0.000146	0.001520	0.000000	0.000000	0.000000	0.000000	0.000000	0.000000	0.000000	0.024264
	B500_a_ave_0-10yrBefore	89.7	0.000342	0.002629	0.000000	0.000000	0.000000	0.000000	0.000000	0.000000	0.000311	0.035225
	B500_a_ave_0-5yrBefore	89.7	0.000482	0.003618	0.000000	0.000000	0.000000	0.000000	0.000000	0.000000	0.000519	0.052326
	B500_a_ave_0-5yrAfter	88.4	0.000584	0.004198	0.000000	0.000000	0.000000	0.000000	0.000000	0.000000	0.001248	0.064973
	B01_a_ave_15-20yrBefore	90.1	0.000225	0.002022	0.000000	0.000000	0.000000	0.000000	0.000000	0.000000	0.000000	0.027533
	B01_a_ave_10-15yrBefore	91.6	0.000227	0.002135	0.000000	0.000000	0.000000	0.000000	0.000000	0.000000	0.000008	0.041416
	B01_a_ave_5-10yrBefore	89.2	0.000245	0.002213	0.000000	0.000000	0.000000	0.000000	0.000000	0.000000	0.000070	0.036217
	B01_a_ave_0-5yrBefore	80.5	0.000662	0.004340	0.000000	0.000000	0.000000	0.000000	0.000000	0.000174	0.001007	0.056797
	B01_a_ave_10-20yrBefore	90.8	0.000267	0.002066	0.000000	0.000000	0.000000	0.000000	0.000000	0.000000	0.000052	0.029667
	B01_a_ave_0-10yrBefore	80.1	0.000449	0.002964	0.000000	0.000000	0.000000	0.000000	0.000000	0.000111	0.000675	0.031544
	B01_a_ave_10-20yrBefore	90.8	0.000267	0.002066	0.000000	0.000000	0.000000	0.000000	0.000000	0.000000	0.000052	0.029667
	B01_a_ave_0-10yrBefore	80.1	0.000449	0.002964	0.000000	0.000000	0.000000	0.000000	0.000000	0.000111	0.000675	0.031544
	B01_a_ave_0-5yrAfter	78.6	0.000775	0.004572	0.000000	0.000000	0.000000	0.000000	0.000000	0.000577	0.002263	0.051625
	B05_a_ave_15-20yrBefore	67.1	0.000167	0.000645	0.000000	0.000000	0.000000	0.000000	0.000003	0.000239	0.001134	0.006039
	B05_a_ave_10-15yrBefore	66.4	0.000221	0.000954	0.000000	0.000000	0.000000	0.000000	0.000007	0.000387	0.001165	0.011634
	B05_a_ave_5-10yrBefore	54.3	0.000227	0.001239	0.000000	0.000000	0.000000	0.000000	0.000021	0.000368	0.000904	0.022414
	B05_a_ave_0-5yrBefore	41.4	0.000532	0.002730	0.000000	0.000000	0.000000	0.000007	0.000135	0.001064	0.002662	0.054618
	B05_a_ave_10-20yrBefore	63.0	0.000194	0.000758	0.000000	0.000000	0.000000	0.000000	0.000012	0.000357	0.001210	0.008835
	B05_a_ave_0-10yrBefore	38.4	0.000377	0.001939	0.000000	0.000000	0.000000	0.000006	0.000105	0.000748	0.001801	0.038397
	B05_a_ave_0-5yrAfter	33.7	0.000643	0.003122	0.000000	0.000000	0.000000	0.000033	0.000259	0.001362	0.003416	0.063699
	B10_a_ave_15-20yrBefore	37.5	0.000228	0.000894	0.000000	0.000000	0.000000	0.000002	0.000041	0.000636	0.001282	0.012478
	B10_a_ave_10-15yrBefore	37.3	0.000259	0.001104	0.000000	0.000000	0.000000	0.000002	0.000054	0.000685	0.001287	0.013949
	B10_a_ave_5-10yrBefore	26.2	0.000359	0.001888	0.000000	0.000000	0.000000	0.000007	0.000097	0.000651	0.001163	0.032033
	B10_a_ave_0-5yrBefore	14.9	0.000680	0.003384	0.000000	0.000000	0.000003	0.000046	0.000332	0.001155	0.001989	0.059630
	B10_a_ave_10-20yrBefore	31.8	0.000243	0.000958	0.000000	0.000000	0.000000	0.000005	0.000070	0.000683	0.001253	0.011885
	B10_a_ave_0-10yrBefore	12.8	0.000516	0.002580	0.000000	0.000000	0.000002	0.000035	0.000225	0.000935	0.001477	0.045730
	B10_a_ave_0-5yrAfter	7.9	0.000784	0.004030	0.000000	0.000001	0.000012	0.000095	0.000452	0.001211	0.001842	0.071674

Table S2B. Descriptive statistics for cyanobacteria concentrations for five buffer sizes, six exposure windows and three concentration metrics. Otherwise as Table S2A.

Type	Variable	%zero	Mean	SD	Min	Q10	Q25	Q50	Q75	Q90	Q95	Max
**Water**	B250_W_ave_15-20yrBefore	94.3	0.000014	0.000242	0.000000	0.000000	0.000000	0.000000	0.000000	0.000000	0.000000	0.005379
**Average**	B250_W_ave_10-15yrBefore	96.1	0.000059	0.000892	0.000000	0.000000	0.000000	0.000000	0.000000	0.000000	0.000000	0.018856
	B250_W_ave_5-10yrBefore	96.4	0.000166	0.002625	0.000000	0.000000	0.000000	0.000000	0.000000	0.000000	0.000000	0.051182
	B250_W_ave_0-5yrBefore	96.2	0.000400	0.004368	0.000000	0.000000	0.000000	0.000000	0.000000	0.000000	0.000000	0.073492
	B250_W_ave_10-20yrBefore	96.4	0.000074	0.001373	0.000000	0.000000	0.000000	0.000000	0.000000	0.000000	0.000000	0.031948
	B250_W_ave_0-10yrBefore	96.2	0.000286	0.002958	0.000000	0.000000	0.000000	0.000000	0.000000	0.000000	0.000000	0.048040
	B250_W_ave_0-5yrAfter	95.5	0.000252	0.002222	0.000000	0.000000	0.000000	0.000000	0.000000	0.000000	0.000000	0.033048
	B500_W_ave_15-20yrBefore	93.2	0.000297	0.004125	0.000000	0.000000	0.000000	0.000000	0.000000	0.000000	0.000000	0.079295
	B500_W_ave_10-15yrBefore	94.7	0.000477	0.005667	0.000000	0.000000	0.000000	0.000000	0.000000	0.000000	0.000000	0.100533
	B500_W_ave_5-10yrBefore	93.8	0.000760	0.007936	0.000000	0.000000	0.000000	0.000000	0.000000	0.000000	0.000000	0.115774
	B500_W_ave_10-20yrBefore	95.0	0.000441	0.004509	0.000000	0.000000	0.000000	0.000000	0.000000	0.000000	0.000000	0.073987
	B500_W_ave_0-10yrBefore	89.7	0.001386	0.010955	0.000000	0.000000	0.000000	0.000000	0.000000	0.000000	0.001598	0.158515
	B500_W_ave_0-5yrBefore	89.7	0.002005	0.015747	0.000000	0.000000	0.000000	0.000000	0.000000	0.000000	0.002787	0.235103
	B500_W_ave_0-5yrAfter	88.4	0.002448	0.018306	0.000000	0.000000	0.000000	0.000000	0.000000	0.000000	0.005827	0.292377
	B01_W_ave_15-20yrBefore	90.1	0.001560	0.017673	0.000000	0.000000	0.000000	0.000000	0.000000	0.000000	0.000000	0.320365
	B01_W_ave_10-15yrBefore	91.6	0.000859	0.007605	0.000000	0.000000	0.000000	0.000000	0.000000	0.000000	0.000025	0.115242
	B01_W_ave_5-10yrBefore	89.2	0.000735	0.006157	0.000000	0.000000	0.000000	0.000000	0.000000	0.000000	0.000288	0.086102
	B01_W_ave_0-5yrBefore	80.5	0.002310	0.014110	0.000000	0.000000	0.000000	0.000000	0.000000	0.001188	0.003955	0.182737
	B01_W_ave_10-20yrBefore	90.8	0.001441	0.014216	0.000000	0.000000	0.000000	0.000000	0.000000	0.000000	0.000294	0.267742
	B01_W_ave_0-10yrBefore	80.1	0.001502	0.009367	0.000000	0.000000	0.000000	0.000000	0.000000	0.000767	0.002551	0.121511
	B01_W_ave_10-20yrBefore	90.8	0.001441	0.014216	0.000000	0.000000	0.000000	0.000000	0.000000	0.000000	0.000294	0.267742
	B01_W_ave_0-10yrBefore	80.1	0.001502	0.009367	0.000000	0.000000	0.000000	0.000000	0.000000	0.000767	0.002551	0.121511
	B01_W_ave_0-5yrAfter	78.6	0.002686	0.014883	0.000000	0.000000	0.000000	0.000000	0.000000	0.003139	0.008079	0.216650
	B05_W_ave_15-20yrBefore	67.1	0.003382	0.017242	0.000000	0.000000	0.000000	0.000000	0.000011	0.001586	0.011023	0.247641
	B05_W_ave_10-15yrBefore	66.4	0.003020	0.014309	0.000000	0.000000	0.000000	0.000000	0.000040	0.002026	0.017473	0.214020
	B05_W_ave_5-10yrBefore	54.3	0.003211	0.014443	0.000000	0.000000	0.000000	0.000000	0.000366	0.002999	0.018406	0.227795
	B05_W_ave_0-5yrBefore	41.4	0.006545	0.019269	0.000000	0.000000	0.000000	0.000237	0.002419	0.014621	0.047528	0.158370
	B05_W_ave_10-20yrBefore	63.0	0.003183	0.015172	0.000000	0.000000	0.000000	0.000000	0.000079	0.001923	0.015636	0.229463
	B05_W_ave_0-10yrBefore	38.4	0.004852	0.015398	0.000000	0.000000	0.000000	0.000186	0.001685	0.011734	0.031603	0.179326
	B05_W_ave_0-5yrAfter	33.7	0.007748	0.020141	0.000000	0.000000	0.000000	0.001223	0.005542	0.015650	0.045038	0.237811
	B10_W_ave_15-20yrBefore	37.5	0.005773	0.018166	0.000000	0.000000	0.000000	0.000063	0.001184	0.021474	0.043997	0.264361
	B10_W_ave_10-15yrBefore	37.3	0.005098	0.013311	0.000000	0.000000	0.000000	0.000049	0.001094	0.020663	0.037668	0.143403
	B10_W_ave_5-10yrBefore	26.2	0.005401	0.013561	0.000000	0.000000	0.000000	0.000232	0.001957	0.019050	0.033434	0.103543
	B10_W_ave_0-5yrBefore	14.9	0.010786	0.022708	0.000000	0.000000	0.000157	0.001400	0.006590	0.042461	0.064561	0.176405
	B10_W_ave_10-20yrBefore	31.8	0.005440	0.015149	0.000000	0.000000	0.000000	0.000113	0.001769	0.021674	0.036502	0.203049
	B10_W_ave_0-10yrBefore	12.8	0.008061	0.016750	0.000000	0.000000	0.000179	0.000993	0.004833	0.032765	0.047519	0.128681
	B10_W_ave_0-5yrAfter	7.9	0.011514	0.023440	0.000000	0.000070	0.000983	0.003234	0.009894	0.037377	0.056260	0.207456

Table S2C. Descriptive statistics for cyanobacteria concentrations for five buffer sizes, six exposure windows and three concentration metrics. Otherwise as Table S2A.

Type	Variable	%zero	Mean	SD	Min	Q10	Q25	Q50	Q75	Q90	Q95	Max
**Maximum**	B250_Max_15-20yrBefore	94.3	0.000365	0.006293	0.000000	0.000000	0.000000	0.000000	0.000000	0.000000	0.000000	0.139864
	B250_Max_10-15yrBefore	96.1	0.000663	0.008848	0.000000	0.000000	0.000000	0.000000	0.000000	0.000000	0.000000	0.159983
	B250_Max_5-10yrBefore	96.4	0.002752	0.040384	0.000000	0.000000	0.000000	0.000000	0.000000	0.000000	0.000000	0.696856
	B250_Max_0-5yrBefore	96.2	0.005258	0.046960	0.000000	0.000000	0.000000	0.000000	0.000000	0.000000	0.000000	0.691358
	B250_Max_10-20yrBefore	96.4	0.000724	0.011745	0.000000	0.000000	0.000000	0.000000	0.000000	0.000000	0.000000	0.268931
	B250_Max_0-10yrBefore	96.2	0.004025	0.037395	0.000000	0.000000	0.000000	0.000000	0.000000	0.000000	0.000000	0.626169
	B250_Max_0-5yrAfter	95.5	0.005941	0.048069	0.000000	0.000000	0.000000	0.000000	0.000000	0.000000	0.000000	0.733291
	B500_Max_15-20yrBefore	93.2	0.003880	0.044974	0.000000	0.000000	0.000000	0.000000	0.000000	0.000000	0.000000	0.703927
	B500_Max_10-15yrBefore	94.7	0.005858	0.061391	0.000000	0.000000	0.000000	0.000000	0.000000	0.000000	0.000000	0.895858
	B500_Max_5-10yrBefore	93.8	0.011456	0.104849	0.000000	0.000000	0.000000	0.000000	0.000000	0.000000	0.000000	1.570108
	B500_Max_10-20yrBefore	95.0	0.005312	0.048427	0.000000	0.000000	0.000000	0.000000	0.000000	0.000000	0.000000	0.740738
	B500_Max_0-10yrBefore	89.7	0.018967	0.122064	0.000000	0.000000	0.000000	0.000000	0.000000	0.000000	0.054730	1.450777
	B500_Max_0-5yrBefore	89.7	0.026397	0.153335	0.000000	0.000000	0.000000	0.000000	0.000000	0.000000	0.099839	2.076191
	B500_Max_0-5yrAfter	88.4	0.036636	0.173572	0.000000	0.000000	0.000000	0.000000	0.000000	0.000000	0.220994	1.866116
	B01_Max_15-20yrBefore	90.1	0.017103	0.133751	0.000000	0.000000	0.000000	0.000000	0.000000	0.000000	0.000047	1.636366
	B01_Max_10-15yrBefore	91.6	0.015785	0.104346	0.000000	0.000000	0.000000	0.000000	0.000000	0.000000	0.004328	1.528735
	B01_Max_5-10yrBefore	89.2	0.020480	0.131797	0.000000	0.000000	0.000000	0.000000	0.000000	0.000000	0.027272	1.719049
	B01_Max_0-5yrBefore	80.5	0.052245	0.208473	0.000000	0.000000	0.000000	0.000000	0.000000	0.099381	0.284275	2.231517
	B01_Max_10-20yrBefore	90.8	0.018048	0.114386	0.000000	0.000000	0.000000	0.000000	0.000000	0.000000	0.033753	1.367577
	B01_Max_0-10yrBefore	80.1	0.036242	0.160696	0.000000	0.000000	0.000000	0.000000	0.000000	0.060885	0.182569	1.609834
	B01_Max_10-20yrBefore	90.8	0.018048	0.114386	0.000000	0.000000	0.000000	0.000000	0.000000	0.000000	0.033753	1.367577
	B01_Max_0-10yrBefore	80.1	0.036242	0.160696	0.000000	0.000000	0.000000	0.000000	0.000000	0.060885	0.182569	1.609834
	B01_Max_0-5yrAfter	78.6	0.077689	0.250294	0.000000	0.000000	0.000000	0.000000	0.000000	0.257559	0.460971	2.039626
	B05_Max_15-20yrBefore	67.0	0.109809	0.342605	0.000000	0.000000	0.000000	0.000000	0.017601	0.262655	0.741261	3.289832
	B05_Max_10-15yrBefore	66.6	0.118854	0.340825	0.000000	0.000000	0.000000	0.000000	0.035873	0.345469	0.726757	3.284877
	B05_Max_5-10yrBefore	54.3	0.156294	0.373409	0.000000	0.000000	0.000000	0.000000	0.114785	0.506527	0.894210	3.108936
	B05_Max_0-5yrBefore	41.6	0.307042	0.497207	0.000000	0.000000	0.000000	0.066616	0.386204	0.985739	1.449746	4.292626
	B05_Max_10-20yrBefore	62.8	0.114213	0.324894	0.000000	0.000000	0.000000	0.000000	0.057998	0.326373	0.815105	3.285754
	B05_Max_0-10yrBefore	38.5	0.231760	0.405023	0.000000	0.000000	0.000000	0.046858	0.279762	0.700321	1.185520	3.184564
	B05_Max_0-5yrAfter	33.7	0.421434	0.563483	0.000000	0.000000	0.000000	0.284029	0.578111	1.134666	1.661972	4.830768
	B10_Max_15-20yrBefore	38.2	0.347817	0.597604	0.000000	0.000000	0.000000	0.046443	0.368332	1.412775	1.772198	3.289832
	B10_Max_10-15yrBefore	37.8	0.354279	0.577637	0.000000	0.000000	0.000000	0.063589	0.401601	1.333860	1.766890	3.284877
	B10_Max_5-10yrBefore	26.2	0.413926	0.603873	0.000000	0.000000	0.000000	0.139982	0.578089	1.287086	1.649939	4.322014
	B10_Max_0-5yrBefore	14.9	0.687890	0.724701	0.000000	0.000000	0.110481	0.473168	1.065871	1.719333	2.025132	5.196959
	B10_Max_10-20yrBefore	32.4	0.351463	0.557598	0.000000	0.000000	0.000000	0.084116	0.441603	1.294012	1.756005	3.285754
	B10_Max_0-10yrBefore	12.8	0.550112	0.619897	0.000000	0.000000	0.076168	0.346037	0.778137	1.459284	1.760564	4.310419
	B10_Max_0-5yrAfter	7.9	0.825269	0.703140	0.000000	0.049823	0.353711	0.668575	1.137550	1.782372	2.035523	5.089031

Table S3. Demographic characteristics of ALS study participants.
Number, percentage or median (with interquartile range in parentheses).

**Variable**	**Cases**, N = 309
Age (Onset/Consent; Years)	63 (55-70)
Sex	
Female	133 (43%)
Male	176 (57%)
Ethnicity	
Hispanic or Latino	2 (0.7%)
Not Hispanic or Latino	305 (99%)
Missing	2
Race	
Asian	1 (0.3%)
Black	4 (1.3%)
Other	1 (0.3%)
White	302 (98%)
Missing	1
Military Service	
Enlisted	41 (13%)
None	268 (87%)
Education	
High school or less	86 (28%)
Bachelor’s degree	70 (23%)
Some postsecondary	98 (32%)
Graduate degree	55 (18%)
Missing	0
Family history of ALS	32 (11%)
Missing	6
Age at Diagnosis (years)	64 (57-71)
ALS Clinical Phenotype	
ALS	279 (90%)
ALS with frontotemporal dementia	16 (5.2%)
Brachial amyotrophic diplegia	9 (2.9%)
Flail limb	2 (0.6%)
Progressive muscular atrophy	3 (1.0%)
El Escorial criteria	
Definite	84 (29%)
Probable	87 (30%)
Probable, lab supported	76 (26%)
Possible	44 (15%)
Suspected	0 (0%)
Missing	18
Onset segment	
Bulbar	84 (27%)
Cervical	105 (34%)
Lumbar	113 (37%)
Respiratory	3 (1.0%)
Thoracic	2 (0.7%)
Missing	2
ALS functional rating scale-revised	37.0 (33.0-41.0)
Missing	2
Time Between Symptom Onset and Diagnosis (years)	1.04 (0.65-1.84)
Status	
Censored	40 (13%)
Observed Death	269 (87%)
Follow up Time from Diagnosis	2.32 (1.41-3.45)
Censored	4.80 (2.71-6.15)
Observed Death	2.12 (1.32-3.22)

Table S4. Unadjusted Cox proportional hazards model results for ALS survival time since diagnosis in years for 5 exposure windows, 5 buffer radii, and 4 exposure metrics. N=307.

Exposure Window (Year)	Buffer Radius (km)	Water Average Concentration			Area Average Concentration			Maximum Concentration			Area Max Concentration		
		HR (95th CI)	P-value	(BH)	HR (95th CI)	P-value	(BH)	HR (95th CI)	P-value	(BH)	HR (95th CI)	P-value	(BH)
0 - 5	0.5	1.08 (0.96, 1.22)	0.189	0.189	1.08 (0.96, 1.21)	0.201	0.201	1.10 (0.97, 1.24)	0.128	0.128	1.10 (0.97, 1.23)	0.132	0.154
years	1.0	1.09 (0.97, 1.22)	0.157	0.189	1.09 (0.97, 1.22)	0.149	0.186	1.10 (0.98, 1.24)	0.110	0.128	1.09 (0.97, 1.22)	0.154	0.154
before	2.5	1.11 (0.99, 1.24)	0.076	0.126	1.14 (1.01, 1.28)	**0.032**	0.054	1.15 (1.02, 1.30)	**0.019**	**0.049**	1.13 (1.00, 1.27)	**0.043**	0.108
onset	5.0	1.21 (1.07, 1.36)	**0.002**	**0.010**	1.22 (1.08, 1.37)	**0.001**	**0.007**	1.22 (1.08, 1.38)	**0.001**	**0.007**	1.20 (1.06, 1.35)	**0.003**	**0.017**
	10.0	1.14 (1.01, 1.29)	**0.028**	0.071	1.15 (1.02, 1.30)	**0.019**	**0.047**	1.14 (1.01, 1.29)	**0.030**	0.050	1.11 (0.99, 1.25)	0.078	0.131
0 - 10	0.5	1.08 (0.97, 1.20)	0.171	0.214	1.07 (0.96, 1.19)	0.216	0.270	1.07 (0.97, 1.20)	0.186	0.186	1.07 (0.97, 1.19)	0.190	0.190
years	1.0	1.09 (0.97, 1.22)	0.168	0.214	1.09 (0.97, 1.23)	0.150	0.250	1.10 (0.97, 1.23)	0.129	0.162	1.10 (0.97, 1.23)	0.131	0.164
before	2.5	1.13 (1.00, 1.27)	**0.043**	0.107	1.12 (1.00, 1.26)	0.055	0.137	1.14 (1.01, 1.28)	**0.028**	0.070	1.14 (1.01, 1.28)	**0.029**	0.072
onset	5.0	1.20 (1.06, 1.35)	**0.003**	**0.014**	1.19 (1.06, 1.34)	**0.004**	**0.020**	1.21 (1.08, 1.37)	**0.002**	**0.008**	1.22 (1.08, 1.38)	**0.001**	**0.006**
	10.0	1.05 (0.93, 1.18)	0.430	0.430	1.07 (0.95, 1.20)	0.292	0.292	1.11 (0.98, 1.25)	0.091	0.152	1.11 (0.98, 1.25)	0.101	0.164
10 - 20	0.5	1.09 (0.97, 1.22)	0.163	0.189	1.08 (0.96, 1.22)	0.177	0.177	1.10 (0.98, 1.24)	0.113	0.117	1.10 (0.98, 1.24)	0.115	0.172
years	1.0	1.10 (0.98, 1.24)	0.096	0.160	1.10 (0.98, 1.24)	0.100	0.166	1.11 (0.98, 1.24)	0.095	0.117	1.09 (0.97, 1.23)	0.137	0.172
before	2.5	1.15 (1.02, 1.29)	**0.020**	0.050	1.16 (1.03, 1.31)	**0.013**	**0.031**	1.18 (1.05, 1.33)	**0.007**	**0.018**	1.17 (1.04, 1.32)	**0.012**	**0.029**
onset	5.0	1.20 (1.07, 1.36)	**0.002**	**0.011**	1.21 (1.08, 1.36)	**0.002**	**0.008**	1.24 (1.09, 1.40)	**<0.001**	**0.004**	1.22 (1.08, 1.38)	**0.001**	**0.007**
	10.0	1.08 (0.96, 1.22)	0.189	0.189	1.09 (0.97, 1.23)	0.135	0.169	1.10 (0.98, 1.24)	0.117	0.117	1.08 (0.96, 1.21)	0.219	0.219
0 - 20	0.5	1.08 (0.96, 1.22)	0.189	0.189	1.08 (0.96, 1.21)	0.201	0.201	1.10 (0.97, 1.24)	0.128	0.128	1.10 (0.97, 1.23)	0.132	0.154
years	1.0	1.09 (0.97, 1.22)	0.157	0.189	1.09 (0.97, 1.22)	0.149	0.186	1.10 (0.98, 1.24)	0.110	0.128	1.09 (0.97, 1.22)	0.154	0.154
before	2.5	1.11 (0.99, 1.24)	0.076	0.126	1.14 (1.01, 1.28)	**0.032**	0.054	1.15 (1.02, 1.30)	**0.019**	**0.049**	1.13 (1.00, 1.27)	**0.043**	0.108
onset	5.0	1.21 (1.07, 1.36)	**0.002**	**0.010**	1.22 (1.08, 1.37)	**0.001**	**0.007**	1.22 (1.08, 1.38)	**0.001**	**0.007**	1.20 (1.06, 1.35)	**0.003**	**0.017**
	10.0	1.14 (1.01, 1.29)	**0.028**	0.071	1.15 (1.02, 1.30)	**0.019**	**0.047**	1.14 (1.01, 1.29)	**0.030**	0.050	1.11 (0.99, 1.25)	0.078	0.131
0 - 5	0.5	0.98 (0.87, 1.12)	0.793	0.885	0.98 (0.87, 1.12)	0.793	0.839	0.99 (0.87, 1.12)	0.818	0.834	0.98 (0.86, 1.12)	0.804	0.804
years	1.0	0.99 (0.87, 1.12)	0.885	0.885	0.99 (0.87, 1.12)	0.839	0.839	0.99 (0.87, 1.12)	0.834	0.834	0.98 (0.86, 1.11)	0.698	0.804
after	2.5	1.04 (0.92, 1.17)	0.579	0.885	1.05 (0.93, 1.19)	0.388	0.646	1.07 (0.94, 1.21)	0.301	0.502	1.05 (0.93, 1.19)	0.449	0.748
onset	5.0	1.09 (0.96, 1.24)	0.172	0.429	1.11 (0.98, 1.26)	0.101	0.252	1.10 (0.97, 1.24)	0.147	0.367	1.07 (0.95, 1.22)	0.257	0.643
	10.0	1.11 (0.98, 1.25)	0.096	0.429	1.13 (1.00, 1.27)	0.055	0.252	1.12 (0.99, 1.27)	0.064	0.321	1.09 (0.97, 1.23)	0.145	0.643

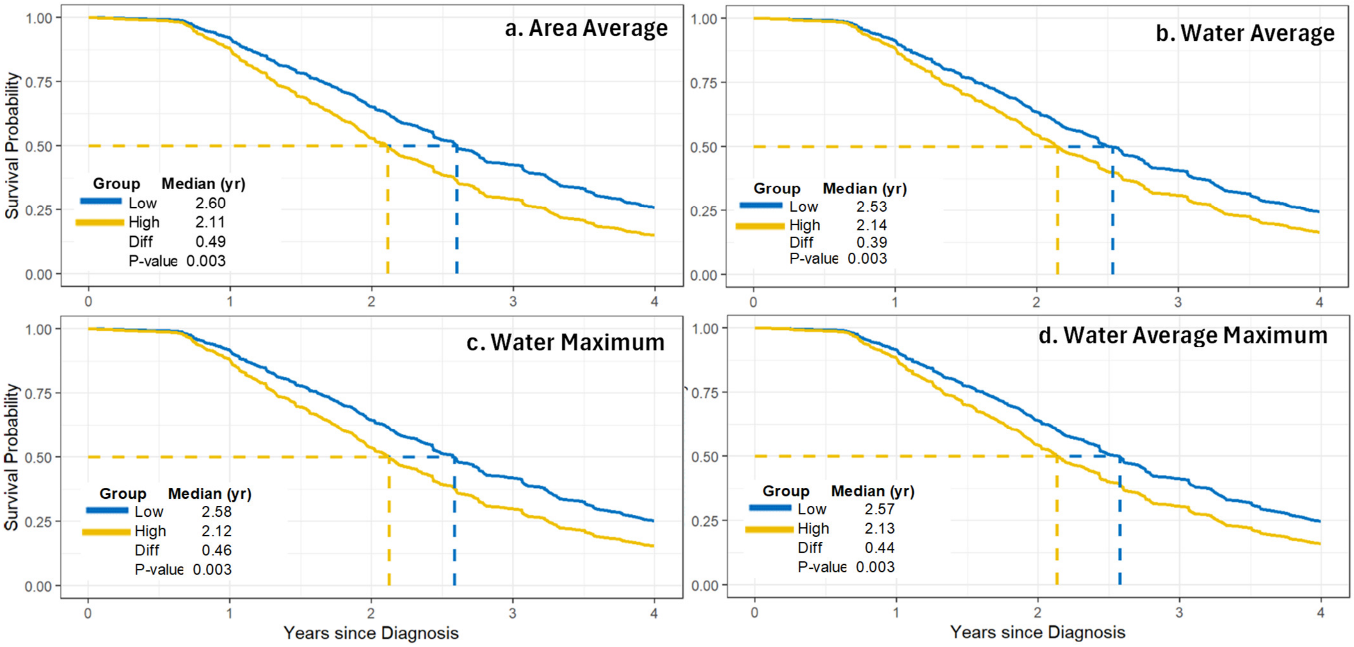

Figure S4. Survival curves for four concentration metrics and low and high exposure groups.
Each curve uses the 0-20 year before onset exposure window, the 5 km buffer, and covariate adjusted Cox models. Median survival given for each group and for difference. N=303.

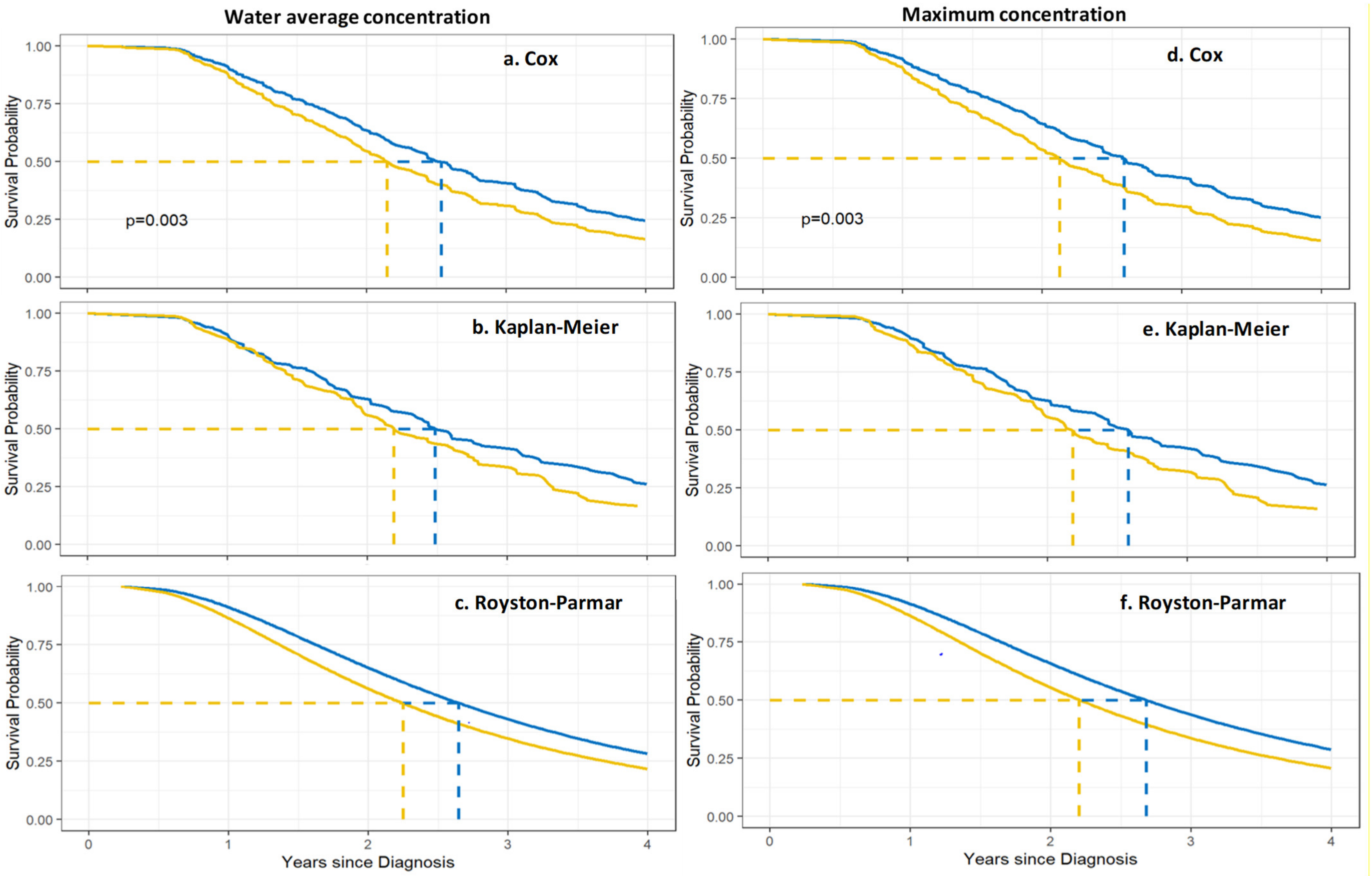

Figure S5. Cox, Kaplan-Meier, and Royston Parmer survival curves.
Compares upper and lower half exposure groups using the 0-20 year exposure window, 5 km buffer, covariate-adjusted models, and two concentration metrics, the water average concentration (left) and the maximum concentration (right). N=303.

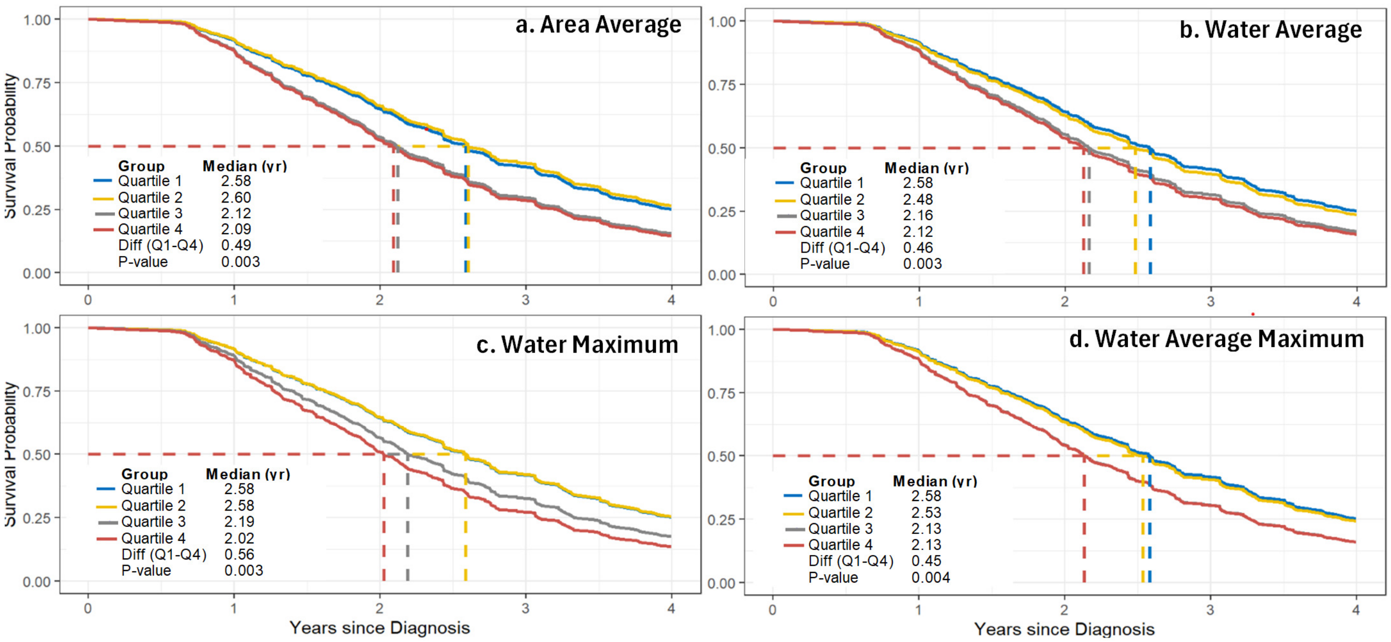

Figure S6. Survival curves for the 0-20 year exposure window using exposure quartiles.
Each curve uses the 0-20 year before onset exposure window, 5 km buffer, and covariate-adjusted Cox models. Median survival given for each group and for difference between first and fourth quartiles. N=303.

Table S5. Cox proportional hazards model results for ALS survival time (years) since diagnosis stratified by private well or city water supply.

Shows results for 7 exposure windows, 4 concentration metrics, and the 5 km buffer. % Zero indicate percentage of individuals without any HAB exposure. Covariates include age at diagnosis, sex, time between onset and diagnosis, onset segment, initial ALSFRS-R, and initial El-Escorial. Excludes participants with other and PLS diagnoses. Model is $Y \sim X\beta_1 + \mathbf{Z}\boldsymbol{\gamma}_1$ for individuals with a private well and $Y \sim X\beta_0 + \mathbf{Z}\boldsymbol{\gamma}_0$ for individuals with city water supply, where X = HAB measure; β_1 = estimated hazard ratio for private well; β_0 = estimated hazard ratio for public water supply, $\mathbf{Z}$ = adjustment covariates. $\boldsymbol{\gamma}$ = estimated coefficients. N=95 for private well and N=141 for public water supply.

Expo-sure Type	Exposure Window (Year)	Buffer Size (km)	Private Well HR (95th CI)	Private Well P-value	Private Well Q-value (BH)	Private Well % Zero	City Water Supply HR (95th CI)	City Water Supply P-value	City Water Supply Q-value (BH)	City Water Supply % Zero
max	0_10	5	1.47 (1.13, 1.90)	**0.004**	**0.020**	33.0%	1.15 (0.95, 1.40)	0.161	0.268	49.3%
max	10_20	5	1.31 (1.04, 1.65)	**0.024**	0.094	60.8%	1.20 (1.01, 1.43)	**0.042**	0.211	71.9%
max	0_20	5	1.58 (1.22, 2.05)	<0.001	**0.002**	27.8%	1.16 (0.95, 1.40)	0.136	0.257	46.8%
max	0_5	5	1.43 (1.10, 1.85)	**0.007**	**0.035**	35.1%	1.13 (0.93, 1.38)	0.213	0.340	50.0%
max	5_10	5	1.35 (1.06, 1.73)	**0.014**	0.072	45.4%	1.18 (0.99, 1.42)	0.070	0.140	62.6%
max	10_15	5	1.22 (0.97, 1.54)	0.094	0.343	63.9%	1.17 (0.99, 1.40)	0.070	0.352	73.0%
max	15_20	5	1.28 (1.01, 1.62)	**0.042**	0.150	67.4%	1.12 (0.94, 1.33)	0.219	0.609	75.6%
a_ave	0_10	5	1.36 (1.07, 1.73)	**0.013**	0.064	33.0%	1.18 (0.98, 1.42)	0.077	0.219	49.3%
a_ave	10_20	5	1.28 (1.02, 1.61)	**0.035**	0.150	60.8%	1.16 (0.98, 1.37)	0.081	0.403	72.7%
a_ave	0_20	5	1.48 (1.16, 1.88)	**0.001**	**0.007**	27.8%	1.18 (0.98, 1.42)	0.073	0.260	46.8%
a_ave	0_5	5	1.35 (1.05, 1.73)	**0.019**	0.093	35.1%	1.16 (0.96, 1.41)	0.116	0.316	50.0%
a_ave	5_10	5	1.30 (1.03, 1.63)	**0.028**	0.142	45.4%	1.18 (0.99, 1.41)	0.069	0.171	62.6%
a_ave	10_15	5	1.19 (0.95, 1.49)	0.128	0.482	63.9%	1.14 (0.96, 1.34)	0.136	0.591	73.0%
a_ave	15_20	5	1.29 (1.01, 1.63)	**0.039**	0.165	66.3%	1.08 (0.91, 1.29)	0.352	0.787	76.3%
w_ave	0_10	5	1.46 (1.13, 1.88)	**0.004**	**0.019**	33.0%	1.16 (0.96, 1.41)	0.131	0.218	49.3%
w_ave	10_20	5	1.30 (1.03, 1.63)	**0.027**	0.078	60.8%	1.17 (0.99, 1.39)	0.073	0.363	72.7%
w_ave	0_20	5	1.59 (1.23, 2.06)	<0.001	**0.002**	27.8%	1.16 (0.95, 1.40)	0.141	0.249	46.8%
w_ave	0_5	5	1.42 (1.10, 1.83)	**0.007**	**0.036**	35.1%	1.15 (0.95, 1.39)	0.163	0.294	50.0%
w_ave	5_10	5	1.33 (1.05, 1.69)	**0.020**	0.100	45.4%	1.17 (0.98, 1.41)	0.088	0.146	62.6%
w_ave	10_15	5	1.20 (0.96, 1.51)	0.105	0.378	63.9%	1.14 (0.96, 1.36)	0.128	0.553	73.0%
w_ave	15_20	5	1.29 (1.02, 1.64)	**0.035**	0.133	66.3%	1.09 (0.92, 1.30)	0.322	0.625	76.3%
amax	0_10	5	1.47 (1.13, 1.90)	**0.004**	**0.018**	33.0%	1.13 (0.93, 1.38)	0.212	0.299	49.3%
amax	10_20	5	1.32 (1.04, 1.66)	**0.022**	0.103	60.8%	1.20 (1.01, 1.43)	**0.040**	0.202	71.9%
amax	0_20	5	1.59 (1.23, 2.06)	<0.001	**0.002**	27.8%	1.14 (0.94, 1.38)	0.189	0.248	46.8%
amax	0_5	5	1.42 (1.11, 1.83)	**0.006**	**0.030**	35.1%	1.11 (0.91, 1.35)	0.304	0.380	50.0%
amax	5_10	5	1.34 (1.05, 1.71)	**0.018**	0.090	45.4%	1.18 (0.98, 1.42)	0.083	0.139	62.6%
amax	10_15	5	1.23 (0.98, 1.56)	0.078	0.372	63.9%	1.19 (0.99, 1.41)	0.060	0.298	73.0%
amax	15_20	5	1.28 (1.01, 1.63)	**0.042**	0.188	67.4%	1.12 (0.94, 1.34)	0.209	0.529	75.6%

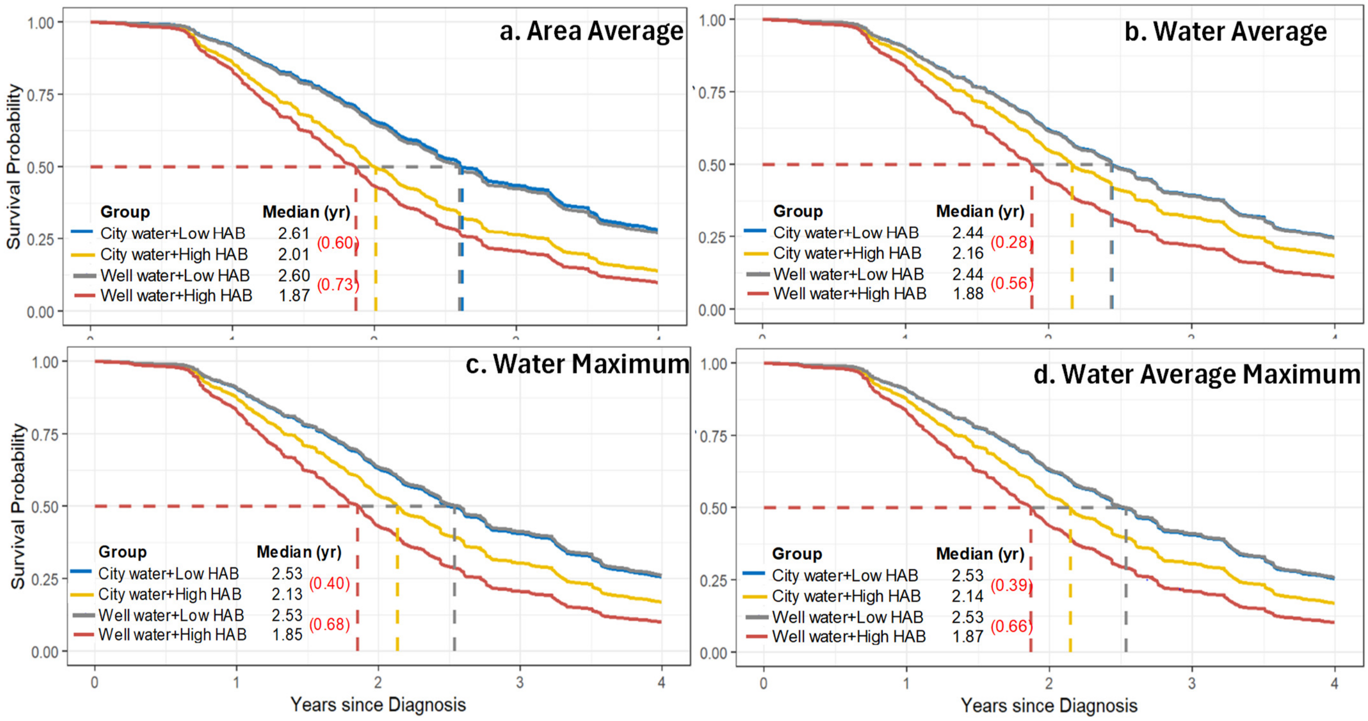

Figure S7. Survival curves comparing upper and lower exposure groups for interactions with water source. Covariate-adjusted exposure using the 5 km buffer and 0-20 year exposure window, and 4 concentration metrics. Median survival given for each group and for difference (in parentheses). N=303.

Table S6. Cox proportional hazards model results for ALS survival time (years) since diagnosis stratified by fishing/swimming.

% Zero indicate percentage of individuals without any HAB exposure. Covariates include age at diagnosis, sex, time between onset and diagnosis, onset segment, initial ALSFRS-R, and initial El-Escorial. Excludes participants with other and PLS diagnoses. Model is Y ~ X β_1 + **Z** $\boldsymbol{\gamma_1}$ for individuals reporting fishing/swimming and Y ~ X β_0 + **Z** $\boldsymbol{\gamma_0}$ for individuals not reporting fishing/swimming, where X = HAB measure; β_1 = estimated hazard ratio for fishing/swimming; β_0 = estimated hazard ratio for not swimming/fishing, **Z** = adjustment covariates. $\boldsymbol{\gamma}$ = estimated coefficients. N=156 for fishing/swimming and N=87 for not fishing/swimming.

Expo-sure Type	Exposure Window (Year)	Buffer Size (km)	Private Well HR (95th CI)	Private Well P-value	Private Well Q-value (BH)	Private Well % Zero	City Water Supply HR (95th CI)	City Water Supply P-value	City Water Supply Q-value (BH)	City Water Supply % Zero
max	0_10	5	1.30 (1.08, 1.56)	**0.005**	**0.016**	45.5%	1.07 (0.84, 1.36)	0.599	0.971	35.6%
max	10_20	5	1.26 (1.06, 1.50)	**0.010**	**0.048**	67.5%	1.18 (0.93, 1.49)	0.179	0.374	65.9%
max	0_20	5	1.34 (1.12, 1.60)	**0.001**	**0.006**	42.9%	1.09 (0.86, 1.38)	0.488	0.766	31.8%
max	0_5	5	1.28 (1.06, 1.53)	**0.008**	**0.042**	48.1%	1.07 (0.84, 1.36)	0.604	0.924	35.6%
max	5_10	5	1.27 (1.06, 1.51)	**0.008**	**0.040**	58.7%	1.17 (0.92, 1.49)	0.202	0.299	48.3%
max	10_15	5	1.20 (1.01, 1.43)	**0.041**	0.203	69.9%	1.13 (0.90, 1.43)	0.288	0.720	66.7%
max	15_20	5	1.20 (1.01, 1.42)	**0.042**	0.210	72.7%	1.12 (0.88, 1.43)	0.341	0.509	70.6%
a_ave	0_10	5	1.28 (1.07, 1.52)	**0.006**	**0.026**	45.5%	1.08 (0.85, 1.38)	0.507	0.958	35.6%
a_ave	10_20	5	1.23 (1.04, 1.46)	**0.018**	0.090	68.2%	1.15 (0.91, 1.45)	0.231	0.413	65.9%
a_ave	0_20	5	1.33 (1.12, 1.58)	**0.001**	**0.007**	42.9%	1.10 (0.87, 1.40)	0.418	0.908	31.8%
a_ave	0_5	5	1.27 (1.06, 1.51)	**0.009**	**0.046**	48.1%	1.07 (0.84, 1.37)	0.572	0.977	35.6%
a_ave	5_10	5	1.24 (1.04, 1.47)	**0.014**	0.070	58.7%	1.16 (0.91, 1.47)	0.226	0.376	48.3%
a_ave	10_15	5	1.17 (0.99, 1.39)	0.073	0.367	69.9%	1.11 (0.89, 1.39)	0.365	0.767	66.7%
a_ave	15_20	5	1.17 (0.98, 1.38)	0.075	0.376	72.7%	1.11 (0.88, 1.41)	0.371	0.529	70.6%
w_ave	0_10	5	1.29 (1.08, 1.54)	**0.005**	**0.027**	45.5%	1.10 (0.86, 1.40)	0.453	0.864	35.6%
w_ave	10_20	5	1.24 (1.04, 1.47)	**0.014**	0.070	68.2%	1.17 (0.92, 1.48)	0.199	0.430	65.9%
w_ave	0_20	5	1.34 (1.12, 1.60)	**0.002**	**0.008**	42.9%	1.10 (0.86, 1.39)	0.457	0.875	31.8%
w_ave	0_5	5	1.27 (1.06, 1.53)	**0.009**	**0.043**	48.1%	1.09 (0.86, 1.39)	0.465	0.817	35.6%
w_ave	5_10	5	1.25 (1.05, 1.49)	**0.011**	0.057	58.7%	1.16 (0.91, 1.47)	0.238	0.372	48.3%
w_ave	10_15	5	1.17 (0.99, 1.39)	0.070	0.260	69.9%	1.13 (0.90, 1.42)	0.297	0.703	66.7%
w_ave	15_20	5	1.18 (0.99, 1.40)	0.063	0.315	72.7%	1.13 (0.89, 1.44)	0.318	0.531	70.6%
amax	0_10	5	1.30 (1.08, 1.56)	**0.005**	**0.019**	45.5%	1.06 (0.83, 1.35)	0.643	0.977	35.6%
amax	10_20	5	1.28 (1.07, 1.52)	**0.007**	**0.034**	67.5%	1.16 (0.92, 1.47)	0.211	0.452	65.9%
amax	0_20	5	1.34 (1.12, 1.60)	**0.002**	**0.008**	42.9%	1.07 (0.84, 1.36)	0.575	0.803	31.8%
amax	0_5	5	1.26 (1.05, 1.51)	**0.011**	0.056	48.1%	1.07 (0.83, 1.36)	0.610	0.875	35.6%
amax	5_10	5	1.28 (1.07, 1.53)	**0.006**	**0.031**	58.7%	1.15 (0.90, 1.46)	0.268	0.335	48.3%
amax	10_15	5	1.22 (1.02, 1.46)	**0.026**	0.130	69.9%	1.13 (0.90, 1.43)	0.300	0.751	66.7%
amax	15_20	5	1.21 (1.02, 1.44)	**0.033**	0.163	72.7%	1.11 (0.87, 1.41)	0.410	0.513	70.6%

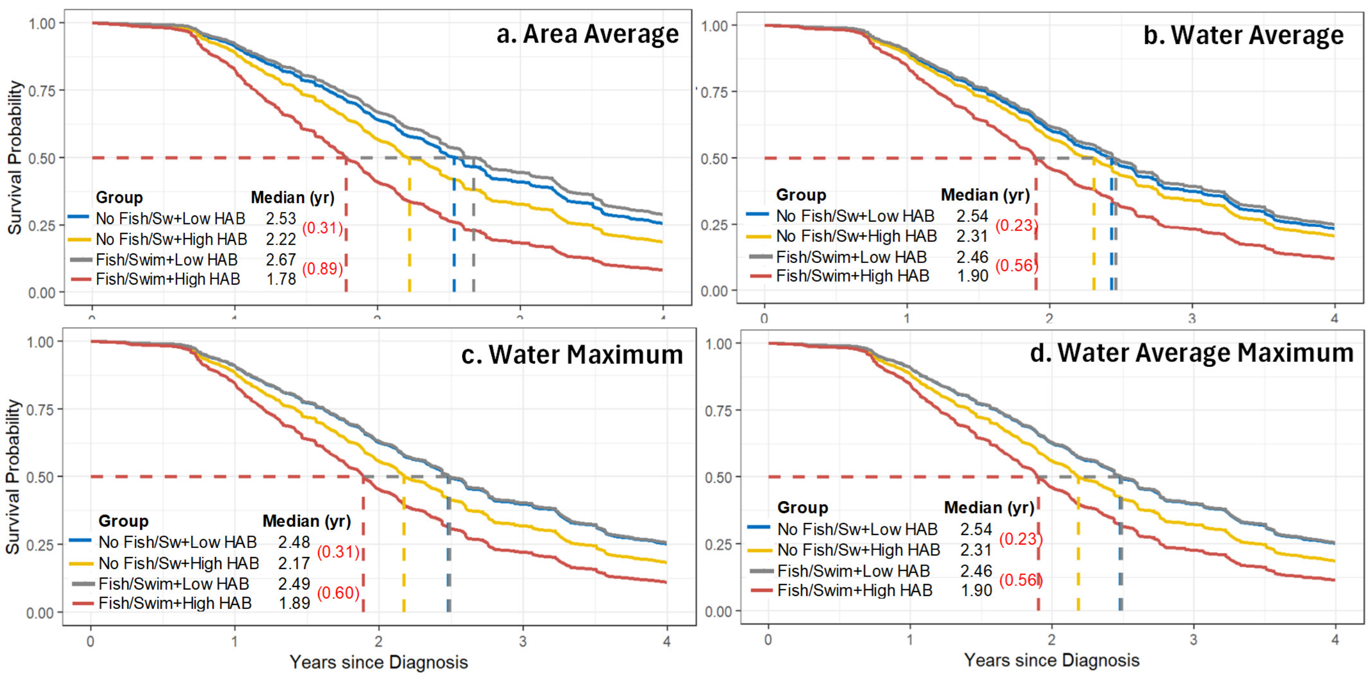

Figure S8. Survival curves comparing upper and lower exposure groups for interactions with fishing and swimming. Covariate-adjusted exposure using the 5 km buffer and 0-20 year exposure window, and 4 concentration metrics. Median survival given for each group and for difference (in parentheses). N=303.
